# Sub-aerial biofilms on urban stone surfaces: community structure and microbial co-occurrence networks

**DOI:** 10.3389/fmicb.2026.1904600

**Published:** 2026-10-22

**Authors:** Magdalena Owczarek-Kościelniak, Alicja Okrasińska, Julia Pawłowska

**Affiliations:** 1 Section for Mycology and Toxicology, Norwegian Veterinary Institute (NVI), Ås, Norway; 2 W. Szafer Institute of Botany, Polish Academy of Sciences, Kraków, Poland; 3 Institute of Evolutionary Biology, Uniwersytet Warszawski Wydzial Biologii, Warsaw, Poland

**Keywords:** algae, bacteria, biodeteriation, cultural heritage, fungi, metabarcoding

## Abstract

Sub-aerial biofilms (SABs) play a key role in the biodeterioration of stone cultural heritage, yet their microbial composition may vary depending on the colonized lithic substrate. The aim of this study was to analyze the taxonomic composition of SABs in relation to the type of stone substrate. The material consisted of ten SAB samples, including eight collected from a historic cemetery in Kraków and two from retaining walls within the city. Sampling was performed using a low-invasive method directly from stone surfaces. Microbial community composition was assessed using Illumina eDNA metabarcoding targeting fungi, algae, and bacteria. Fungal communities were dominated by representatives of the classes *Dothideomycetes* and *Eurotiomycetes*. Among phototrophic organisms, *Chlorophyta* constituted the majority of algal taxa, while cyanobacteria were also highly represented. Bacterial diversity was largely composed of *Proteobacteria* and *Actinobacteria*, which together accounted for approximately 60% of the detected bacterial taxa. The overall taxonomic composition of SAB communities was consistent with results reported previously using culture-dependent approaches. These findings confirm the suitability of using eDNA metabarcoding for comprehensive characterization of SABs and highlight substrate-related patterns in microbial colonization of stone surfaces. Co-occurrence network analysis identified contrasting interaction patterns among microbial domains, with highly integrated fungal communities, more modular bacterial assemblages, and fragmented phototrophic networks, suggesting that the stability of SAB is driven not only by taxonomic composition but also by domain-specific patterns of microbial interactions.

## Introduction

Stone-built structures, decorative elements, and works of art, while possessing undeniable aesthetic, cultural, and historical value, also constitute habitats for highly specialized microbial communities. Regardless of climatic conditions, stone substrates represent extremely inhospitable environments, characterized by intense solar radiation, pronounced temperature fluctuations, limited water and nutrient availability, and elevated salinity or electrolyte concentrations. These abiotic constraints, together with biotic interactions among microorganisms, strongly shape the structure, composition, and functioning of microbial communities colonizing stone surfaces (Gorbushina, 2007).

Despite these harsh conditions, lithic materials are successfully colonized by diverse assemblages of stress-tolerant microorganisms, including bacteria, cyanobacteria, algae, and fungi (De Leo et al., 2022; Gulotta et al., 2018; Ortega-Morales et al., 2019; Prieto et al., 2024). At the stone-air interface, these organisms form structured microbial consortia known as sub-aerial biofilms (SABs). SABs are embedded in a self-produced extracellular polymeric substance (EPS) matrix composed primarily of polysaccharides, proteins, and extracellular DNA, which facilitates adhesion to the substrate, provides protection against environmental stressors, and enables metabolic interactions among different microbial groups (Tenore et al., 2023; Soares et al., 2025; Liu et al., 2022).

SABs play a significant role in various ecological and applied contexts, particularly in the conservation of built heritage (Liu et al., 2022; Bendia et al., 2021; Trovão et al., 2019; Trovão et al., 2020; Paiva et al., 2022). Their impact on stone materials is complex and often ambivalent. On one hand, SABs may contribute to bioprotection by forming a physical and chemical barrier that reduces the direct impact of environmental factors such as solar radiation, temperature fluctuations, and moisture loss. On the other hand, they can actively participate in biodeterioration processes through biological weathering mechanisms, including organic and inorganic acid production, chelation, mineral dissolution, and mechanical damage resulting from hyphal penetration and biofilm expansion. Over time, these processes may lead to substantial structural and aesthetic degradation of stone substrates (Yadav and Purchase, 2025).

Beyond their interaction with built surfaces, SABs also play a role in atmospheric and urban biogeochemical processes. At larger spatial scales, they can contribute to carbon sequestration and reduction of atmospheric CO₂ concentrations, participate in nitrogen cycling through processes such as atmospheric nitrogen fixation, and act as sinks for airborne pollutants and heavy metals, particularly in urban and industrial environments (Bendia et al., 2021; Prieto et al., 2020; Vázquez-Nion et al., 2020; Gianini et al., 2025; Dehkohneh et al., 2026; Sterflinger and Krumbein, 1995; Ortega-Morales et al., 2000). These processes are primarily discussed at broader ecosystem scale, however they are ongoing in the build environment, thus highlighting the ecological relevance of SABs beyond stone biodeterioration.

Although the role of biological factors in stone deterioration has been increasingly recognized, existing research is predominantly focused on artworks and architectural structures located in warmer climates. Considerably less attention has been paid to temperate and colder regions, as well as to specific heritage settings such as historic cemeteries, which often contain stone monuments of high cultural, historical, and artistic value (Owczarek-Kościelniak et al., 2020; Kärnefelt et al., 2014; Matwiejuk, 2008; Matwiejuk, 2009). Moreover, many studies concentrate on individual taxonomic groups rather than on whole microbial communities, thereby overlooking inter-taxa interactions that may significantly influence biofilm development, stability, and deteriorative potential.

The aim of this study was to characterize the taxonomic composition of fungal, algal, and bacterial communities forming sub-aerial biofilms on stone substrates of different types using DNA metabarcoding, and to assess differences in community structure in relation to the colonized lithic material. Additionally, microbial co-occurrence patterns were analyzed to explore potential inter-taxa relationships within SAB communities.

## Materials and methods

### Stone sampling

Stone sampling was conducted at the Rakowicki Cemetery, a cultural heritage site located in Kraków, southern Poland. Superficial stone material was collected from gravestones using sterile needle scraping under maximally aseptic conditions. For each gravestone, four surface areas measuring approximately 1.5 × 1.5 cm were sampled from different aspects (orientations) at a height of approximately 1.5 m above ground level. In total, four limestone, one dolomite, and three sandstone gravestones were sampled. Due to the limited representation of dolomite gravestones at the Rakowicki Cemetery, two of the dolomite samples were collected from historic retaining walls located by Władysława Łokietka Street and Kamienna Street in Kraków, increasing the total number of dolomite samples to three. The collected stone powder was transferred into sterile, single-use 2 mL microcentrifuge tubes and stored at −5 °C until further laboratory analyses. Sample metadata is available in Supplementary material A.

### DNA extraction, library preparation and sequencing

DNA was extracted from the collected stone powder using the FastDNA™ Spin Kit for Feces (MP Biomedicals, USA), following the manufacturer’s protocol. The efficiency of DNA extraction and the quality of the obtained DNA were assessed spectrophotometrically using a NanoDrop 2000 instrument (Thermo Scientific, USA). Extracted DNA was suspended in TES buffer provided with the extraction kit and stored in frozen conditions until further analyses.

For high-throughput sequencing, three molecular markers were selected: the fungal ITS2 region, the bacterial 16S rRNA gene, and the bacterial 23S rRNA gene (Table 1). Amplicon libraries were prepared using Phanta Max Super-Fidelity DNA Polymerase (2 × Phanta Max Master Mix; Vazyme, China), following the manufacturer’s recommendations. Prior to library quality and concentration assessment with the NEBNext® Library Quant Kit for Illumina® (New England Biolabs, USA), amplicons were indexed using Nextera XT reagents (Illumina ®, USA). Equimolar pooling of libraries was performed prior to sequencing. High-throughput paired-end sequencing (2 × 250) was carried out on an Illumina MiSeq platform with the Illumina MiSeq Reagent Kit v2 (Illumina ®, USA).

**Table 1:** List of primers used in this study.

Target loci	Primer	Direction	Sequence (5′ → 3′)
ITS	I_ITS3	Forward	GCATCGATGAAGAACGCAGC
	I_ITS4	Backward	TCCTCCGCTTATTGATATGC
16S	16S_319F	Forward	CCTACGGGNGGCWGCAG
	16S_806R	Backward	GACTACHVGGGTATCTAATCC
23S	23S_p23SrV_f1	Forward	GGACAGAAAGACCCTATGAA
	23S_p23SrV_r1	Backward	TCAGCCTGTTATCCCTAGAG

### Bioinformatic data processing

The quality of raw sequencing reads was assessed using FastQC v0.12.1 (Andrews, 2010) to evaluate base quality scores, length distribution, and the sequencing artifacts presence. Subsequent bioinformatic analyses were performed using the QIIME2 v. 2026.4.0 pipeline (Bolyen et al., 2019). Fungal (ITS2), bacterial (16S rRNA), and phototrophic (23S rRNA) datasets were processed independently.

Demultiplexed paired-end sequencing reads were imported into the QIIME 2 environment and summarized to assess overall sequencing quality. Sequence denoising and amplicon sequence variants (ASVs) inference were performed using the DADA2 algorithm implemented in QIIME 2. Trimming and truncation parameters determined from quality score profiles and applied for low-quality bases removal. Singleton ASVs were removed.

Taxonomic classification of ASVs was performed using the consensus BLAST-based classifier implemented in QIIME 2 (feature-classifier classify-consensus-blast). For fungal ITS2 sequence classification, the UNITE v10 database for QIIME2 (release 2025-02-19) (Abarenkov et al., 2025), for 16S sequence classification - the SILVA SSU 138.2 database (release 2024-07-11) (Quast et al., 2013; Yilmaz et al., 2014), and for the phototrophic microorganisms the 23S rDNA Plastidial Reference μgreen-db r1.1 reference database (release 2019-09-06) (Djemiel et al., 2020). The SILVA database was transformed for QIIME2 as described in the QIIME2 documentation. For the μgreen-db the mothur format was downloaded and formatted using R 4.6.0. Taxonomic assignment was conducted with a minimum sequence identity threshold of 97%.

Taxonomic composition was visualized using stacked bar plots to verify consistency across samples and datasets. Subsequently, ASVs classified as *Unassigned* were excluded from downstream analyses. Additionally, from the 16S dataset sequences classified as Cyanobacteria were excluded. Both feature tables and representative sequences were filtered accordingly. Downstream analyses and visualizations were performed in R 4.6.0, using packages *qiime2R* (v0.99.6) (Bisanz, 2018), *tidyverse* (Wickham et al., 2019), ggplot2 (Wickham, 2016), and viridis (Garnier et al., 2024).

Community compositions were summarized independently for fungi, bacteria and phototrophic organisms. The most abundant taxa were identified based on total relative abundance across all samples. Taxa classified as *incertae sedis* or of unresolved placement were retained and reported, as they constituted a nonnegligible fraction of the assemblage.

Heatmaps of relative abundances were generated for selected taxonomic ranks using log₁₀-transformed relative abundance data with a small pseudocount to avoid undefined values. Only the most abundant taxa were visualized to improve interpretability.

### Diversity and differential abundance

Alpha- and beta-diversity analyses were performed using QIIME 2 core diversity metrics at marker-specific rarefaction depths. Diversity comparisons among substrate types were assessed using Shannon diversity indices, and Bray-Curtis and Jaccard distance matrices. Group differences were tested using PERMANOVA and PERMDISP with 999 permutations.

Differential abundance analyses across substrates were conducted using ANCOM-BC2 as implemented in QIIME 2 (Lin and Peddada, 2024). Substrate type was included as a fixed effect, dolomite was used as the reference level, Benjamini-Hochberg correction was applied for multiple testing, and structural zeros and prevalence filtering were enabled to improve robustness. Resulting differentially abundant taxa were visualized using the ANCOM-BC2 visualizer.

### Co-occurrence networks

Co-occurrence patterns of all the datasets were explored in *R*, using the NetCoMi package (Peschel et al., 2020). Taxa were aggregated to the genus level, based on taxonomic annotations, to reduce sparsity. Duplicate genus names originating from different higher-rank taxa were disambiguated by appending numeric identifiers. Data were transformed using a centered log-ratio (CLR) transformation, and zero counts were handled using multiplicative replacement (multRepl). Pairwise associations between taxa were estimated using Spearman rank correlations.

To reduce network complexity and focus on strong associations, sparsification was performed using a correlation threshold approach, retaining only correlations with an absolute value ≥0.6. A global co-occurrence networks were constructed, followed by substrate-specific networks built separately for dolomite, limestone, and sandstone samples. Community structure was assessed using the cluster_fast_greedy algorithm. Central taxa (hubs) were identified based on degree centrality and eigenvector centrality. Additional visualization performed via the qgraph backend implemented in NetCoMi. Presence-absence patterns at the genus level were additionally verified using genus-collapsed feature tables generated in QIIME 2.

## Results

ITS2 amplicons were successfully obtained from all samples. In contrast, amplification of the 16S rRNA gene failed for one dolomite (sample B) and one sandstone sample (sample 9), while amplification of the 23S rRNA gene was unsuccessful for two dolomite (samples B and K) and one sandstone samples (sample 9) (Supplementary material A).

High-quality datasets were obtained for fungi, bacteria, and phototrophic microorganisms after quality filtering, removal of singleton features, and exclusion of Unassigned reads. All three datasets were characterized by uneven feature abundance distributions, with a limited number of dominant taxa coexisting with numerous low-abundance features. The fungal dataset comprised 10 samples, yielding 151 unique ITS features and a total of 233,737 sequence reads. Sequencing depth per sample was relatively even, with a single exception, ranging from 8,435 to 36,490 reads, with a median of 24340.5 and a mean of 23373.7 reads per sample. Feature abundances were highly skewed, spanning from 3 to 31,850 reads per feature. Following removal of cyanobacterial sequences, the bacterial dataset consisted of 8 samples containing 205 unique features, with a total of 80,957 sequence reads. Sequencing depth per sample ranged from 1,928 to 19,186 reads (median 9,944.5, mean 10119.6). Feature-level abundances ranged from 16 to 2,827 reads per feature. The phototrophic dataset included 7 samples, comprising 78 unique features and a total of 492,692 sequence reads, representing the highest sequencing depth among the analyzed markers. Per-sample read counts ranged from 40,832 to 93,795, with a median of 77,761 and a mean of 70384.6 reads per sample. Feature abundances showed pronounced skewness, ranging from 8 to 96,843 reads per feature.

High-throughput sequencing revealed an uneven taxonomic structure of the fungal community, characterized by strong dominance at all taxonomic levels. At the phylum level, Ascomycota dominated, accounting for 84.0% of total reads, whereas Mucoromycota contributed 15.1%. All remaining phyla - Basidiomycota, Mortierellomycota and Chytridiomycota, collectively represented less than 1% of the community. At the class level, the assemblage was dominated by Eurotiomycetes (28.6%), Lecanoromycetes (24.0%) and Dothideomycetes (20.7%), while Mucoromycetes formed a substantial secondary component (14.9%), while other classes occurred at low relative abundances (<1%) (Figure 1). A notable proportion of reads was assigned to Ascomycota *incertae sedis* (10.4%). Overall, the fungal assemblage was characterized by dominance of a limited number of taxonomic groups across multiple hierarchical levels, with minor contributions from a diverse pool of low-abundance taxa (Figure 2).

**Figure 1:**
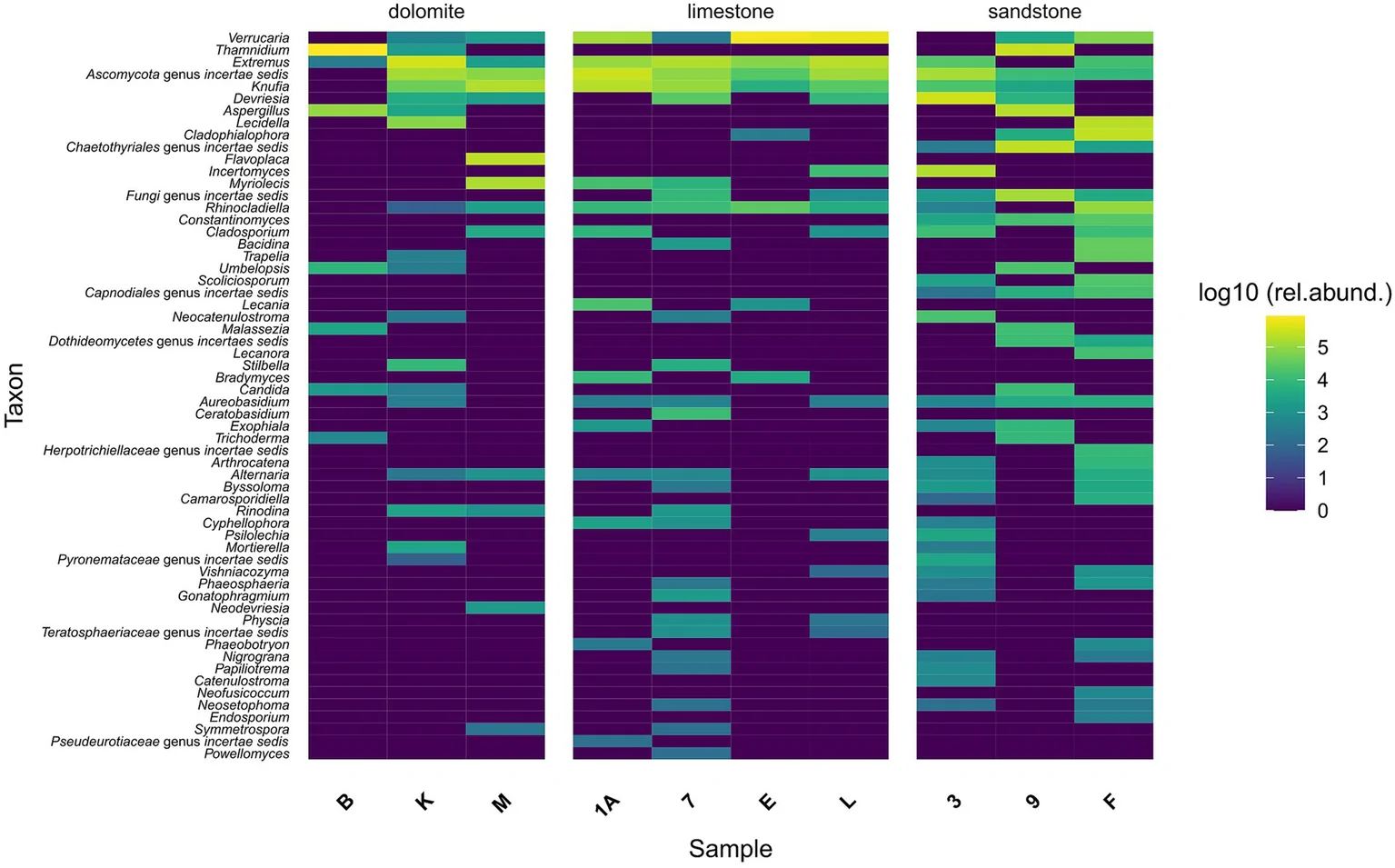
Heatplot of the most abundant fungal genera.

**Figure 2:**
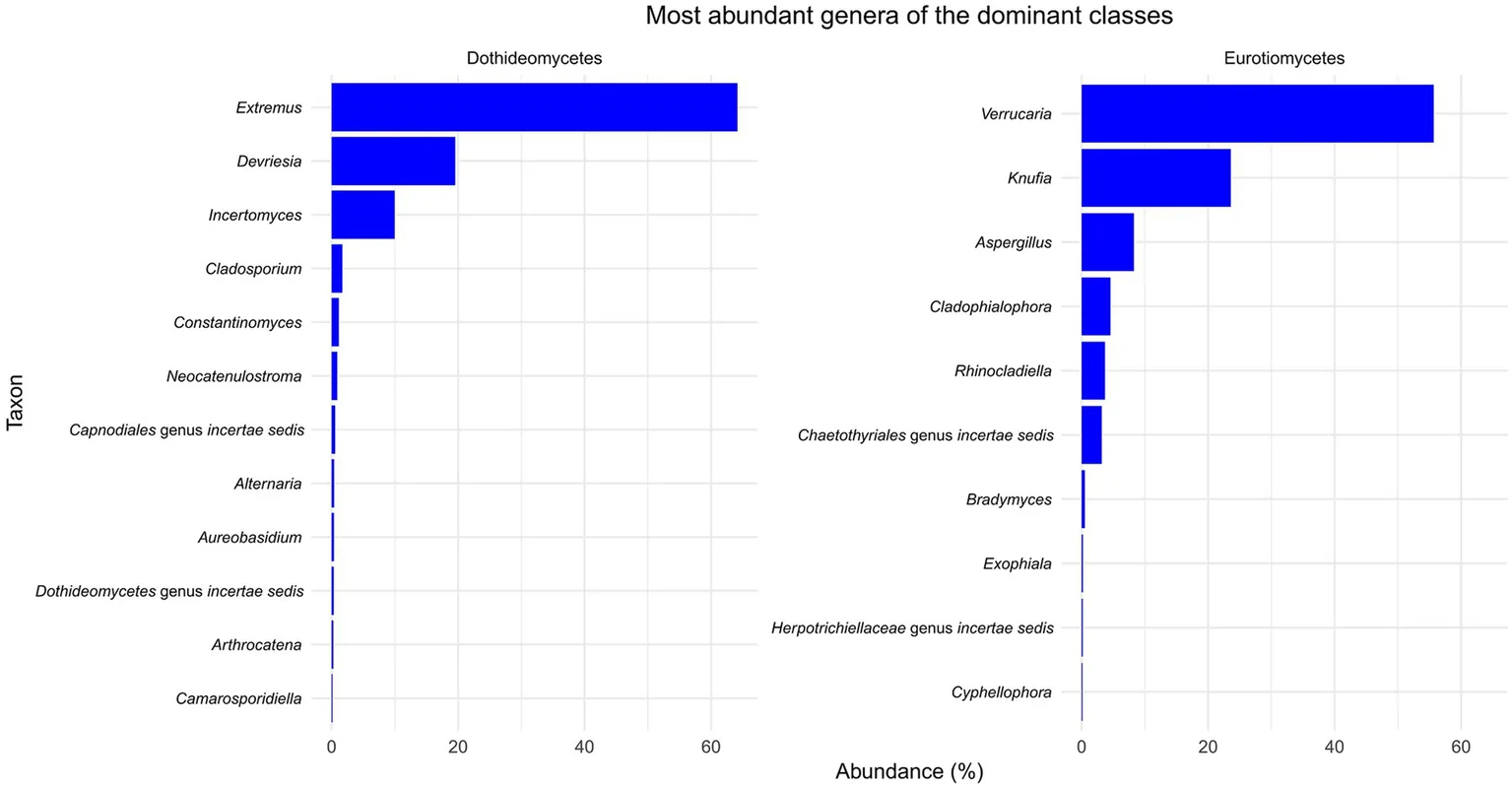
The dominant genera of the most abundant fungal classes.

The bacterial community was dominated by Pseudomonadota (Proteobacteria) (44.0%) and Actinomycetota (Actinobacteria) (40.1%). Secondary phyla included Bacteroidota (8.2%) and Deinococcota (4.4%), while all remaining phyla occurred at low abundances. At the class level, Alphaproteobacteria (44.0%) and Actinobacteria (23.2%) were most abundant, complemented by Rubrobacteria (16.5%) and Bacteroidia (8.2%). The most abundant bacterial orders were Sphingomonadales, Rubrobacterales, Micrococcales, Cytophagales, and Frankiales. Figure 3 presents a heatplot generated for most abundant bacterial genera.

**Figure 3:**
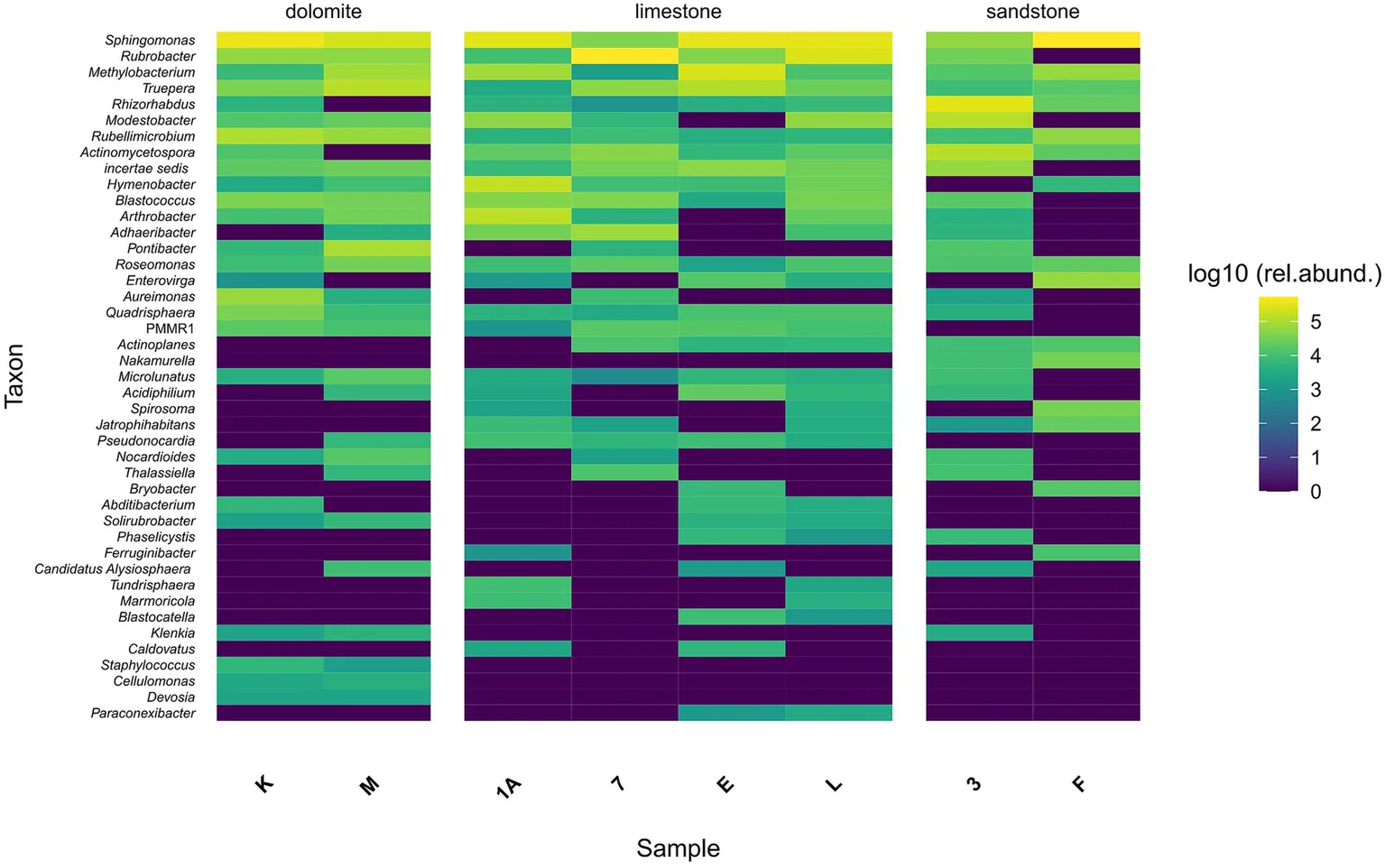
Heatplot of the most abundant bacteria on the genus level.

Phototrophic organisms formed a distinct and abundant component of the community. At the phylum level, eukaryotic green algae (Chlorophyta) dominated (86.2%), while Cyanobacteria accounted for 12.5% of total phototrophic reads. Streptophyta contributed 1.3%, whereas all remaining groups occurred in negligible amounts. At the class level, Trebouxiophyceae clearly prevailed (82.1%), reflecting the dominance of algal lineages commonly involved in epilithic growth and lichen symbioses. Cyanobacterial sequences were largely assigned to unresolved higher ranks, together representing 16.0%. The most abundant orders were Prasiolales, Microthamniales (eukaryotic algae), and Chroococcidiopsidales (cyanobacteria). At the genus level, *Trebouxia* (45.2%) and *Chroococcidiopsis* (44.2%) together accounted for nearly 90% of phototrophic reads (Figure 4). Species-level analysis revealed a strong dominance of uncultured *Chroococcidiopsis* spp. (82.1%), followed by *Prochlorothrix hollandica* and *Klebsormidium flaccidum*. The high proportion of uncultured taxa highlights limited reference coverage for epilithic and subaerial phototrophs.

**Figure 4:**
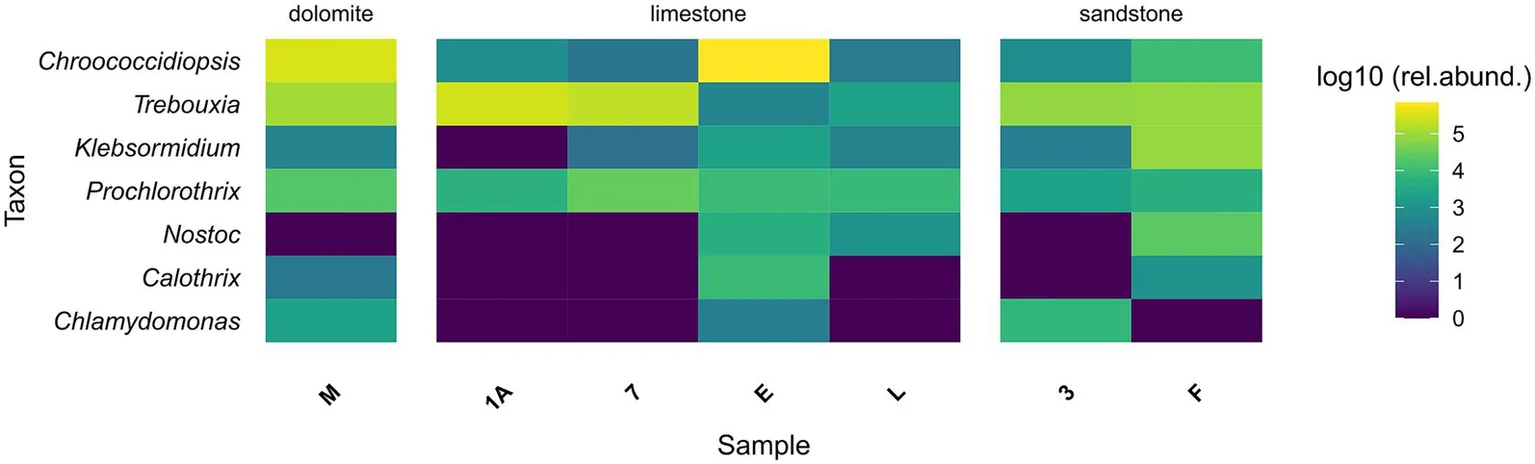
Heatplot of the most abundant phototrophic genera.

Alpha diversity, expressed by the Shannon index, did not differ significantly among stone substrate types (dolomite, limestone, sandstone) for any of the analyzed microbial groups, indicating comparable levels of within-sample diversity across substrate type. In terms of beta diversity, fungal and phototrophic community composition showed no significant differences among substrate types using either presence-absence-based (Jaccard) or abundance-based (Bray-Curtis) distance metrics. In contrast, bacterial communities exhibited a significant substrate-related structuring, as revealed by both Jaccard- and Bray-Curtis-based PERMANOVA analyses, while multivariate dispersion remained homogeneous among groups. Statistical results are available in Supplementary material B.

Differential abundance analysis performed using ANCOM-BC2 did not identify any taxa with statistically significant differences among stone substrate types for fungi, bacteria, or phototrophic microorganisms after false discovery rate correction (*q* < 0.05).

Co-occurrence network analyses revealed pronounced differences in global network architecture across microbial domains colonizing stone surfaces. Fungal and bacterial communities each formed a single, fully connected network component, whereas phototrophic communities exhibited a fragmented structure composed of three disconnected components, with the largest connected component encompassing approximately 82% of taxa.

The global fungal co-occurrence network showed a single connected component comprising 71 taxa (Figure 5). The network exhibited a high clustering coefficient (0.61) and moderate modularity (0.48), with most associations being positive (approx. 92%). Six clusters were identified, varying in size from 3 to 23 taxa. Most lichenized fungal taxa were grouped into one of three clusters. The cluster 1 (*n* = 23) grouped *Flavoplaca*, *Physcia*, *Psilolechia*, *Rinodina*, *Verrucaria* and not precisely identified *Lecanorales* representative. Another set of lichenized fungi – *Bacidina*, *Byssoloma*, *Lecanora*, *Lecidiella*, *Scoliciosporum* and Trapelia, was placed in cluster 2 (*n* = 16). The cluster 4 contained *Lecania*, *Myriolecis* and not-classified representatives of *Lecanoraceae* and *Teloschistaceae*. Additionally, cluster 5 included a not-classified representative of *Verrucariales*, known for lichenized taxons. Analyses showed that all the identified clusters contained melanized extremotolerant fungi, with four clusters (3, 4, 1 and 2 – in decreasing number of included taxa). Cluster 3 included genera *Aureobasidium*, *Cladophialophora*, *Constantinomyces*, not-classified representatives of families belonging to *Chaetothyriales* and Dothideomycetes, and not-classified genus in family *Dothiceaceae*. In cluster 4 were grouped genera often associated with stone substrate and demanding environments – *Bradymyces*, *Cyphellophora*, *Extremus* and *Knufia*. Other black fungi were *Catenulostroma*, *Devriesia*, *Incertomyces*, *Neodevriesia* and not-classified genera of *Neodevriesiaceae* and *Teratosphaeriaceae* grouped in cluster 1; and *Arthrocatena*, *Rhinocladiella* and not-classified genera of *Herpotrichellaceae* and *Trichomeriaceae* grouped in cluster 2; *Neocatenulostroma* in cluster 5 and *Exophiala* in cluster 6. Saprotrophic fungi, like *Alternaria*, *Aspergillus*, *Ceratobasidium*, *Cladosporium*, *Mortierella*, *Trichoderma*, were identified in all the clusters, with 1, 2 and 6 being the richest. *Catenulostroma*, not-classified genus of Lecanorales, *Phaeobotryon* and unidentified Ascomycetes were indicated as the central hubs of the fungal network. Fungal networks were characterized by substrate-specific dominance patterns, while retaining a conserved pool of highly connected taxa. On limestone, the highest degree centrality was observed for *Byssoloma*, *Neocatenulostroma*, *Neosetophoma*, *Nigrograna*, and *Papiliotrema*. Sandstone networks were centered around *Catenulostroma*, *Bradymyces*, *Ceratobasidium*, and representatives of *Botryosphaeriaceae* and Ascomycota (genus *incertae sedis*). Dolomite networks were dominated by *Arthrocatena*, *Aspergillus*, *Bacidina*, *Bradymyces*, and *Botryosphaeriaceae* (genus *incertae sedis*). Several of these genera (e.g., *Catenulostroma*, *Bradymyces*, *Botryosphaeriaceae*) appeared as global or cluster hubs across multiple substrates. All highly central taxa were present across multiple substrate types.

**Figure 5:**
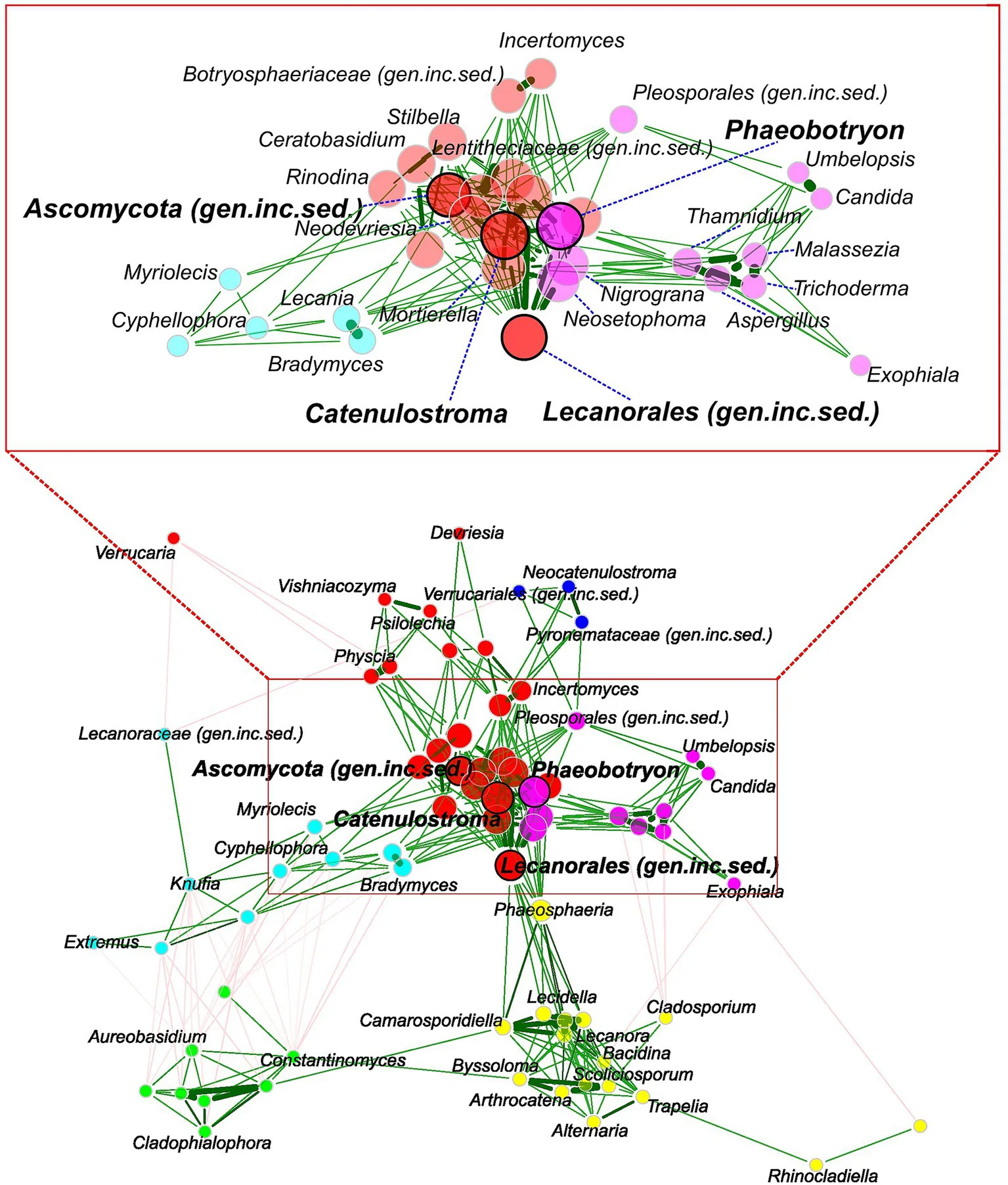
Co-occurrence global network of the fungal genera. Nodes are coloured by cluster affiliation. Node size is proportional to eigenvector centrality. Edges indicate Spearman correlations (|r| ≥ 0.6), with thicker edges representing stronger associations, where green color indicates positive, and red negative correlations. Central hub taxa are marked with a black outline.

Bacterial networks were also fully connected but differed markedly in topology, showing lower clustering, fewer positive associations (~56%), and higher modularity. The global bacterial co-occurrence network consisted of a single connected component comprising 53 genera (Figure 6). The network showed moderate clustering (0.49) and relatively high modularity (0.57), with the lower proportion of positive associations (~56%), then in case of fungal networks. Bacterial networks showed a consistency of hub taxa, particularly among Actinobacteriota. Limestone networks were dominated by *Blastococcus*, *Aureimonas*, *Cellulomonas*, and unclassified *Microbacteriaceae*. In contrast, sandstone and dolomite networks exhibited very high degree centrality values for *Abditibacterium*, *Actinoplanes*, *Blastocatella*, *Actinomycetospora*, and unclassified *Acetobacteraceae*. Several of these taxa (*Abditibacterium*, *Actinoplanes*, *Aureimonas*) recurred as central nodes across substrates. *Blastocatella*, *Enterovirga* and *Klenkia* were indicated as the central network hubs.

**Figure 6:**
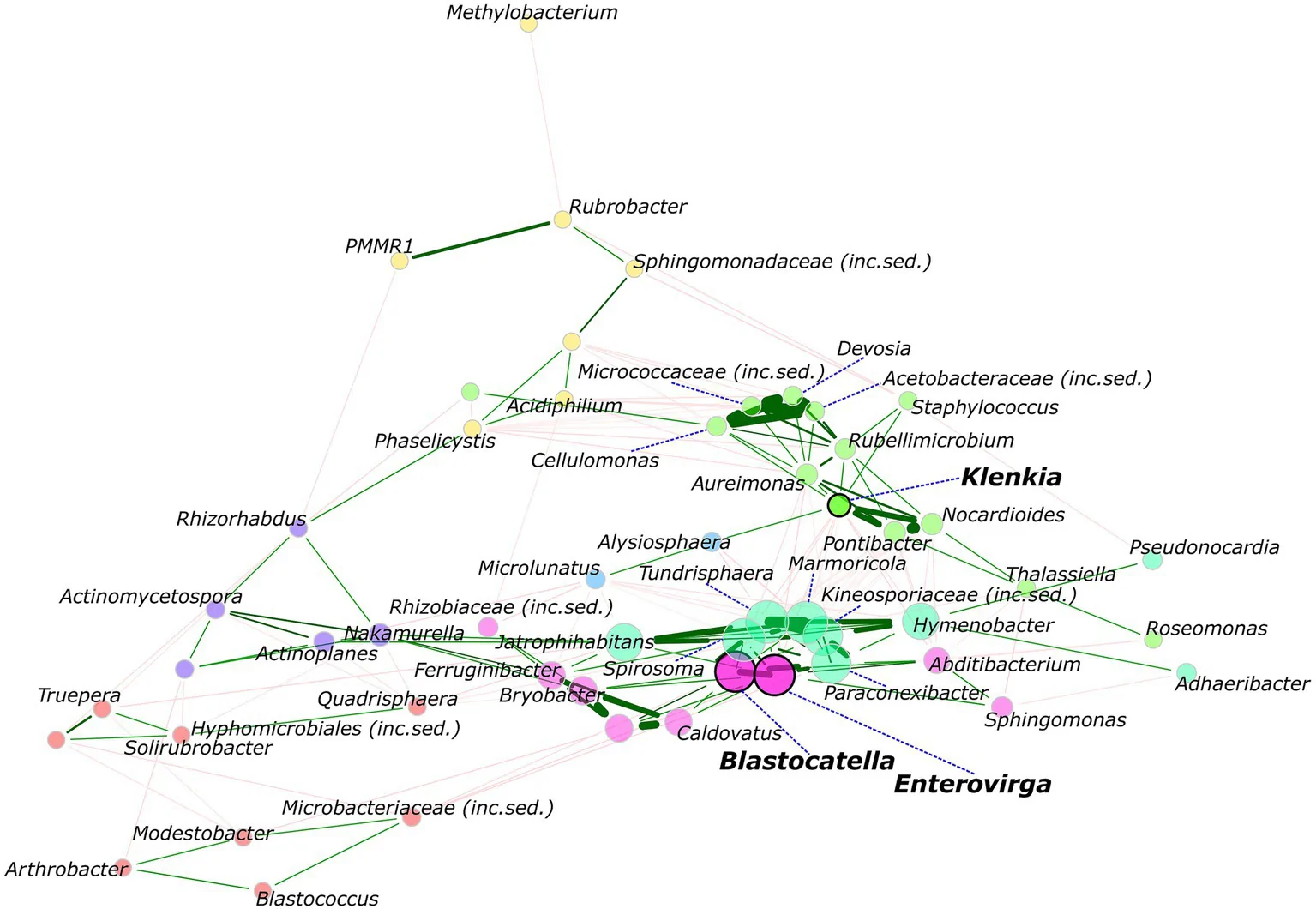
Co-occurrence global network of the bacteria genera. Nodes are coloured by cluster affiliation. Node size is proportional to eigenvector centrality. Edges indicate Spearman correlations (|r| ≥ 0.6), with thicker edges representing stronger associations, where green color indicates positive, and red negative correlations. Central hub taxa are marked with a black outline.

Phototrophic networks showed the weakest structural integration. Their fragmentation into multiple components resulted in undefined average path length at the whole-network level and low betweenness centrality across most taxa. The global phototrophic co-occurrence network comprised 17 taxa distributed across three network components, with the largest connected component (LCC) encompassing approximately 82% of all taxa (Figure 7). The network exhibited moderate clustering (0.42) and relatively high modularity (≈0.57). Approximately 67% of edges within the LCC were positive. Centrality analysis identified a limited number of structurally important taxa. The highest degree and eigenvector centrality values were observed for *Polytrichaceae* (genus *incertae sedis*) – indicated as a network hub, followed by *Trebouxia*, *Chlamydomonas*, *Klebsormidium*, and *Nostoc*.

**Figure 7:**
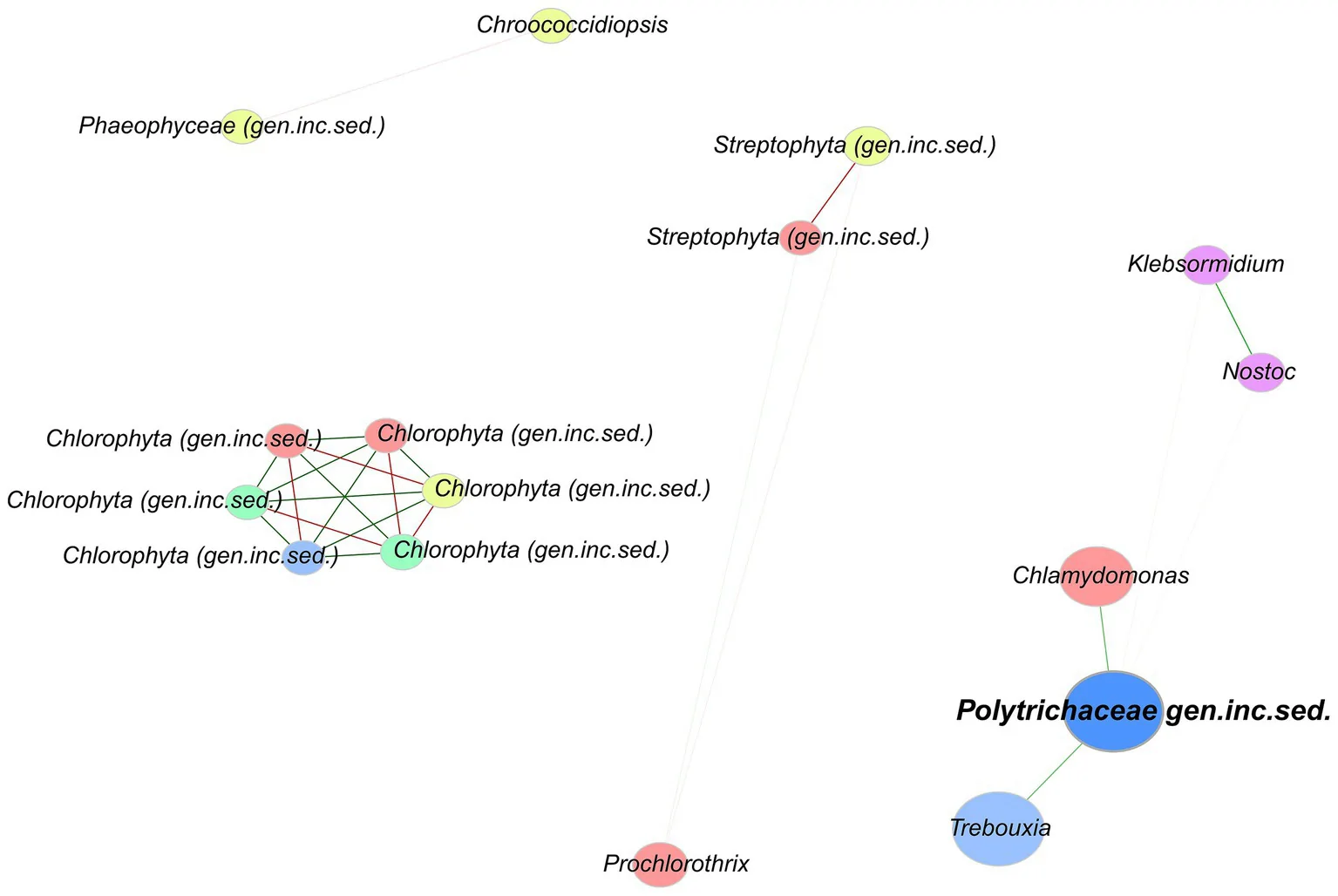
Co-occurrence global network of the algae and cyanobacteria genera. Nodes are coloured by cluster affiliation. Node size is proportional to eigenvector centrality. Edges indicate Spearman correlations (|r| ≥ 0.6), with thicker edges representing stronger associations, where green color indicates positive, and red negative correlations. Central hub taxa are marked with a black outline.

## Discussion

Our study provides one of the first culture-independent, multi-domain characterizations of sub-aerial biofilms (SABs) colonizing stone monuments in temperate climate regions. By integrating fungal, bacterial, and phototrophic datasets with co-occurrence network analyses, our results offer a community-level perspective on lithic biofilms that complements and extends earlier studies (Owczarek-Kościelniak et al., 2020).

Across all datasets, SAB communities exhibited a strongly uneven abundance structure, with a relatively limited number of dominant taxa embedded within a diverse pool of low-abundance lineages. Such distributions are typical for extreme or highly selective environments, and reflect strong environmental filtering imposed by biotic and abiotic factors. The dominance of stress-tolerant taxa supports the view that SABs are shaped primarily by abiotic constraints (Sterflinger and Krumbein, 1995; Louati et al., 2020; Yang et al., 2025).

Fungal communities were overwhelmingly dominated by Ascomycota, particularly Eurotiomycetes, Lecanoromycetes, and Dothideomycetes. These groups comprise many slow-growing, melanized, or otherwise stress-resistant taxa commonly reported from rock, monument, and desert habitats (Gulotta et al., 2018; Trovão et al., 2019; Trovão et al., 2020; Paiva et al., 2022; Paiva et al., 2023; Paiva et al., 2025; Lepinay et al., 2017; Gaylarde et al., 2017a; Isola et al., 2013; Isola et al., 2016; Selbmann et al., 2014; Sert et al., 2007; Ruibal et al., 2009; Ruibal et al., 2008; Friedmann, 1982; Berti et al., 2023). The substantial representation of Lecanoromycetes, together with the dominance of *Trebouxia* among phototrophs, suggests widespread lichen-associated components on the stone surfaces (Muggia et al., 2015; Pląskowska et al., 2024; Pinheiro et al., 2019). Such micro-lichenic assemblages, as stone areas with developed lichen thallus were avoided, may represent early or persistent successional stages before macroscopic thalli become visible (Lepinay et al., 2017). The high proportion of sequences assigned to unresolved fungal lineages underscores the still limited taxonomic resolution of reference databases for epilithic fungi and highlights the likelihood of substantial undescribed diversity in temperate stone habitats.

Phototrophic assemblages were strongly dominated by Trebouxiophyceae green algae and by the cyanobacterial genus Chroococcidiopsis, lineages well known from endolithic and subaerial habitats. These organisms likely play a substantial role in biofilm establishment by providing organic carbon to heterotrophic organisms. The high proportion of uncultured or poorly resolved phototrophic taxa is a direct consequence of the reference sequence collection limitations and highlights the need for building accessible and continuously updated databases. Our results align with established biogeographic patterns, showing that sub-aerial biofilms in temperate climates are typically dominated by green algae, while cyanobacterial dominance is more characteristic of arid environments (Gaylarde and Gaylarde, 2005; Hallmann et al., 2013; Murray et al., 2021).

In contrast to fungi and phototrophs, bacterial communities exhibited statistically significant substrate-related structuring at the beta-diversity level. The predominance of Pseudomonadota and Actinomycetota is consistent with their metabolic versatility, EPS production, and resistance to UV radiation (Gaylarde, 2020). Interestingly, differential abundance analysis did not identify individual taxa significantly associated with specific stone types after multiple-testing correction. This might suggest that substrate effects are expressed primarily through shifts in relative abundances and community-level organization rather than through the presence or absence of taxa. Such subtle restructuring may be sufficient to alter functional properties of biofilms while remaining difficult to detect with univariate approaches, especially given the limited number of samples per substrate type (Weiss et al., 2017). Additionally, interpretation of dolomite-associated communities requires caution, as all dolomite samples originated from objects located outside the cemetery area, thus observed differences might be additionally impacted by local environmental variations. Research on cultural heritage objects, especially gravestones and funerary monuments, is constrained by both conservation and ethical considerations. Thus, the number of samples available for semi-invasive sampling remained relatively small, and the results should be interpreted with appropriate caution. Results comparison with the 2020 study based on morphological and culture-dependent approach reveals both clear overlap and notable discrepancies with the molecularly detected phototrophic assemblages in the present study.

Bacteria are ubiquitous microorganisms, with some genera are well documented from biodeteriorated stone. One of the best described examples is the genus *Rubrobacter*, also shown in our study, leading to pinkish discoloration and participation in crystal formation (Laiz et al., 2009; Schabereiter-Gurtner et al., 2001). Interestingly, most notions of this genus come from thermophilic environments. Several of genera identified in out study were previously reported from biodeteriorated materials – *Acidiphilum*, *Arthrobacter*, *Blastococcus*, *Marmoricola*, *Modestobacter*, *Solirubrobacter* or *Sphingomonas* (Meng et al., 2023; Heyrman et al., 2005; Suchy et al., 2024; Urzì et al., 2001; Essoussi et al., 2010; Rizk et al., 2023; Sbissi et al., 2025; Jroundi et al., 2020; Urzì et al., 2000; Montero-Calasanz et al., 2019; Trujillo et al., 2015; Choe et al., 2021; Gaylarde et al., 2017b; Schröer et al., 2020; Li et al., 2016). Three genera identified in our study as central hub taxa are either known inhabiting stone environments or demanding niches. *Klenkia* is an actinomycete identified as a stone colonizer and highly resistant taxon (Gtari et al., 2012; Sun et al., 2018), *Blastocatella* was shown as a calcium carbonate precipitating extremophilic bacteria, from cave walls and stone monuments (Lange-Enyedi et al., 2024; Li et al., 2018), while *Enterovirga* is a member of the family Methylobacteriaceae, members of which are often associated with efficient biofilm development (Lee et al., 2024).

In both studies, the dominance of green algae (Chlorophyta), typical of lithic and sub-aerial habitats, was observed. *Klebsormidium flaccidum* was identified in both datasets and represents a well-known, stress-tolerant taxon frequently reported from stone surfaces, biological soil crusts, and early successional biofilms (Futó et al., 2024; Gaylarde and Gaylarde, 2005; Hallmann et al., 2013; Hartmann et al., 2020; Karsten and Rindi 2010; Laiz et al., 2009; Schabereiter-Gurtner et al., 2001; Meng et al., 2023). Its recurrence supports its role as a ubiquitous component of sub-aerial phototrophic communities. Several taxa reported in the 2020 study - such as desmids (*Cosmarium* spp.), planktonic or semi-planktonic chlorophytes (*Desmodesmus*, *Scenedesmus*, *Pediastrum*), filamentous green algae (*Oedogonium*), and diatoms (*Hantzschia amphioxys*) - were absent or only marginally represented in the metabarcoding dataset. These organisms are often associated with transient moisture availability, nutrient inputs, or soil contact, and may not form stable, long-term components of sub-aerial biofilms (Hallmann et al., 2013; Gaylarde, 2020; Jung et al., 2025).

Cyanobacterial assemblages reported in the 2020 study were very narrow. Observed taxa (*Oscillatoria* sp., *Tolypothrix fasciculata*) are morphologically conspicuous lithobionts that are readily detected by light microscopy and relatively amenable to cultivation. In contrast, the metabarcoding data were dominated by *Chroococcidiopsis*, a genus absent from the 2020 dataset. This discrepancy is consistent with well-documented methodological bias: *Chroococcidiopsis* is a slow-growing, morphologically simple cyanobacterium that is difficult to culture and can be overlooked in microscopy-based surveys. Those taxa were previously identified from lithic or demanding environments (Baldanta et al., 2023; Heyrman et al., 2005; Suchy et al., 2024). Importantly, interpretations of photobiont diversity based solely on metabarcoding data remain uncertain, as current reference databases for aeroterrestrial algae are still limited; therefore, such results should ideally be supported by classical approaches, including microscopy and cultivation.

The fungal communities show substantial agreement between approaches. Many melanized, rock-inhabiting fungi reported in 2020 - including *Knufia marmoricola*, *Neocatenulostroma* spp., *Aureobasidium pullulans*, and undescribed Teratosphaeriaceae - were also detected molecularly and, in several cases, emerged as highly connected taxa in co-occurrence networks. This concordance confirms their role as structurally and functionally important members of SABs (Isola et al., 2016; Ruibal et al., 2009). Minor differences, such as the detection of additional cryptic or unresolved ascomycete lineages, most likely reflect sensitivity of eDNA-based methods and difficulties in culturing and isolation rather than true ecological absence in the earlier study. Metabarcoding analysis has shown a significant representation of melanized extremophiles on all three types of substrates. Genera *Arthrocatena*, *Constantinomyces*, *Devriesia*, *Extremus*, *Knufia* and *Neodevriesia* include species often associated from stone and/or extreme habitats, including cultural heritage object (Isola et al., 2016; Ruibal et al., 2009; Ruibal et al., 2008; Egidi et al., 2014; Owczarek-Kościelniak, 2020; Owczarek-Kościelniak and Sterflinger, 2018; Ruibal et al., 2005; Ruibal et al., 2018; Sun et al., 2020; Brewer and Fierer, 2018). Several species belonging to genera *Bradymyces*, *Catenulostroma*, *Incertomyces* and *Neocatenulostroma* are also referred to as extremotolerant saxicolous taxa, however they are less frequently reported in the literature. *Cladophialophora*, *Exophiala* and *Rhinocladiella* are genera classified as melanized black yeasts, with species described as opportunistic pathogens and occurring often in polluted environments. Their high participation in the fungal community observed in our study reflects their importance in SAB communities. Importantly, *Catenulostroma*, *Phaeosphaeria*, *Constantinomyces* and *Bradymyces* were indicated as cluster 1, 2, 3 and 4 central hubs, highlighting their role in stone communities.

Several of the dominant and well connected in the network taxa have been repeatedly associated with stone biodeterioration process. The genus *Knufia* is particularly well known for multiple species isolated only from stone substrates – limestone, marble, sandstone and travertine, including objects of cultural heritage (Paiva et al., 2025; Isola et al., 2016; Selbmann et al., 2014; Brewer and Fierer, 2018; Favero-Longo et al., 2011; Sterflinger et al., 1997; Voigt et al., 2020). In recent years multiple new species isolated from stone substrates have been described in this genus, making it possibly the best currently documented and widespread stone biodeteriorating fungus. Genus (*Devriesia*) *Neodevriesia* also includes representatives of stone-inhabiting fungi, forming black microcolonies and penetrafing within the stone substrate (Egidi et al., 2014; Isola et al., 2022; Ruffolo et al., 2017; Toreno et al., 2024). Previously classified within the genus Devriesia, multiple saxicolous fungi were moved to genera Neodevriesia and Extremus. In the genus *Bradymyces* four out of five described up to date species were described from stone environment – *B. graniticola* and *B. alpinus*, *B. pullus*, and *B. yunnanensis*, presenting a preference of this genus towards stone substrate (Sun et al., 2020; Réblová et al., 2016; Hubka et al., 2014). Above mentioned fungi and other melazined microcolonial stone colonizing fungi lead to stone biodeterioration through visual and structural alterations of stone structures. They are involved in formation of black spots and/or patina, surface crumbling, pitting and exfoliation, leading inevitably to loss of original esthetic value of stone objects (De Leo et al., 2022). Fungal biodeteriorative actions can be caused by mechanical interactions, enzymatic activity and pigment deposition, thus involvement of other non-melanized genera should be taken into consideration.

The extensive lichen diversity documented in 2020 - including numerous crustose and foliose species of *Verrucaria*, *Flavoplaca*, *Myriolecis*, *Lepraria*, and *Physcia* - was not directly mirrored in the molecular data. This difference is expected and reflects methodological scope. The metabarcoding approach targeted bulk DNA and focused primarily on ITS2 sequences that may not reliably resolve lichenized fungi to species level (Nilsson et al., 2019). Nevertheless, key lichen-forming fungal groups (e.g., Lecanoromycetes) were abundant in the molecular dataset, indicating that lichenized fungi form an integral component of the broader stone community (Gadd and Dyer, 2017).

In summary, these results indicate that classical and molecular approaches capture different but complementary facets of stone-associated microbial diversity. Their integration supports the view of SABs as stable, multi-kingdom consortia composed of lithobiontic fungi, free-living and lichen-associated phototrophs, and extremotolerant cyanobacteria.

The exploratory co-occurrence network analyses presented in this study, however performed on a limited number of samples, showed domain-specific patterns in the organization of fungal, bacterial and phototrophic communities. The fungal network formed a single connected component, with a predominance of positive associations, indicating a highly integrated community structure. The ocurrence of shared hub taxa points to a conserved fungal “core” potentially contributing to SAB stability. These taxa were represented by melanized extremotolerant taxa (*Catenulostroma*, *Devriesia*, *Extremus*, *Neodevriesia*, *Rhinocladiella*, *Knufia* and *Bradymyces*), followed by well-known lichenized fungi (*Flavoplaca*, *Physcia*, *Rinodina*, *Verrucaria*, *Lecanora*, *Lecidella* and *Trapelia*). Central position of melanized rock-inhabiting or stress-tolerant fungi in clusters suggests their high contribution to SAB community stability. Bacterial networks displayed higher modularity and a greater proportion of negative associations, indicating stronger competitive interactions and niche partitioning. Phototrophic co-occurrence networks were significantly fragmented, with fewer strong associations and lower general integration. Thay may reflect greater niche segregation among phototrophic taxa, driven by variation in light intensity, moisture retention, and surface microtopography (Hallmann et al., 2013; Villa and Cappitelli, 2019), however these variables were not quantified during sampling and their relative contribution to community assembly could not be assessed directly.

Our findings emphasize that SABs on stone monuments constitute integrated, multi-kingdom systems where ecological stability stems from complex interaction networks rather than from isolated taxa. The co-occurrence of lichenized genera and stress-tolerant melanized fungi suggests their joint contribution to SAB development and persistence. Our results suggest that fungi may play an important role in structuring the SAB community, while the fungal-photobiont associations remain an important element of mature stone biofilms.

Importantly, this study addresses a geographic and contextual gap in SAB research by focusing on a temperate-region historic cemetery, an often-overlooked but culturally significant heritage setting. Future work should expand sampling to include factors such as seasonal dynamics, microclimatic measurements, and functional approaches. Such data would enable identification of direct links between community structure, metabolic potential, and stone deterioration or bioprotection processes, ultimately informing more sustainable conservation strategies for stone-built heritage.

Presented findings should be interpreted within the study-specific constraints, including the limited sample number, the location of the dolomite samples, incomplete environmental metadata, and limitations of reference phototrophic taxonomic databases.

## Data Availability

Raw sequencing data generated in this study have been deposited in the NCBI Sequence Read Archive (SRA) under the BioProject accession number PRJNA1538185. Sequence reads are publicly accessible at NCBI SRA via the individual run accessions SRR40984040–SRR40984064.
