# Supplementary material for: Sub-aerial biofilms on urban stone surfaces: community structure and microbial co-occurrence networks
Source: Front Microbiol. 2026 Oct 22;17:1904600. doi: 10.3389/fmicb.2026.1904600 (PMC13736629; doi:10.3389/fmicb.2026.1904600)
Supplement: Supplementary file 1 [file Data_Sheet_1.pdf]

Supplementary materials A.

Sample overview

#	sample	localization	substrate_type	library preparation success		
				ITS	16S	23S
1	**1A**	the Rakowicki Cemetery	limestone	+	+	+
2	**3**	the Rakowicki Cemetery	sandstone	+	+	+
3	**7**	the Rakowicki Cemetery	limestone	+	+	+
4	**9**	the Rakowicki Cemetery	sandstone	+		
5	**B**	the Rakowicki Cemetery	dolomite	+		
6	**E**	the Rakowicki Cemetery	limestone	+	+	+
7	**F**	the Rakowicki Cemetery	sandstone	+	+	+
8	**K**	Kamienna Street	dolomite	+	+	
9	**L**	the Rakowicki Cemetery	limestone	+	+	+
10	**M**	Władysława Łokietka Street	dolomite	+	+	+
			∑=	10	8	7
