# Supplementary material for: Sub-aerial biofilms on urban stone surfaces: community structure and microbial co-occurrence networks
Source: Front Microbiol. 2026 Oct 22;17:1904600. doi: 10.3389/fmicb.2026.1904600 (PMC13736629; doi:10.3389/fmicb.2026.1904600)
Supplement: Supplementary file 2 [file Data_Sheet_2.pdf]

# I. Dataset: fungi

## I.I Shannon index

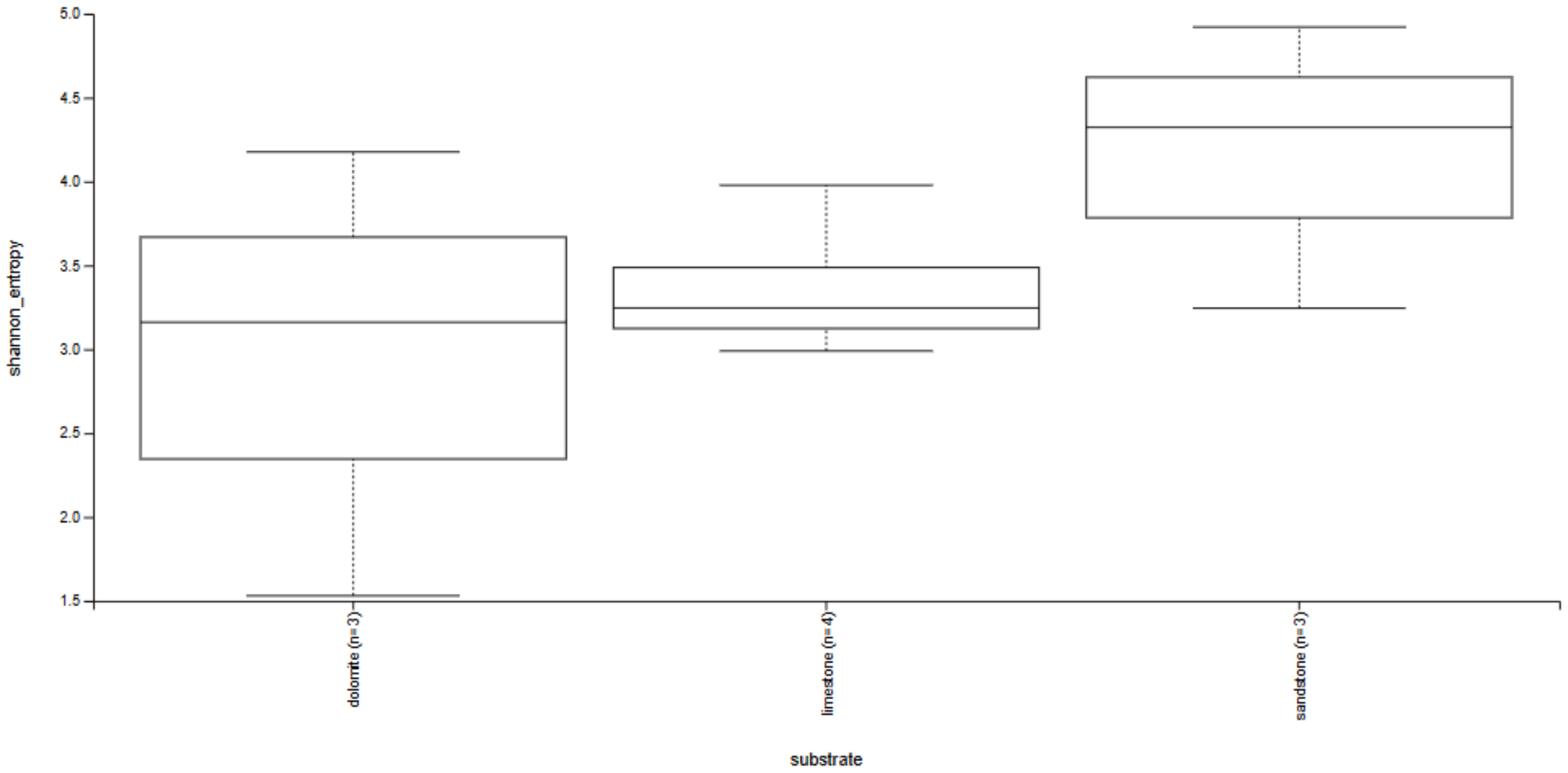


*Figure 1. Shannon diversity of fungal communities across stone substrate types.*

*Tab. 1. Overall comparison of fungal alpha diversity among stone substrate types based on the Shannon diversity index (Kruskal-Wallis test).*

| | Result |
|---|---|
| H | 3.027272727272724 |
| p-value | 0.22010812774168523 |

*Tab. 2. Pairwise comparisons of fungal alpha diversity among stone substrate types based on the Shannon diversity index (Kruskal-Wallis test with FDR correction).*

| | | H | p-value | q-value |
|---|---|---|---|---|
| Group 1 | Group 2 | | | |
| dolomite (n=3) | limestone (n=4) | 0.125000 | 0.723674 | 0.723674 |
| | sandstone (n=3) | 2.333333 | 0.126630 | 0.827259 |
| limestone (n=4) | sandstone (n=3) | 2.000000 | 0.157299 | 0.235949 |

## I.II Bray-Curtis

*Tab. 3. Overall comparison of fungal community composition among stone substrate types based on Bray-Curtis dissimilarity (PERMANOVA).*

| | |
|---|---|
| test statistic name | pseudo-F |
| sample size | 10 |
| number of groups | 3 |
| test statistic | 1.316545 |
| p-value | 0.198 |
| number of permutations | 999 |

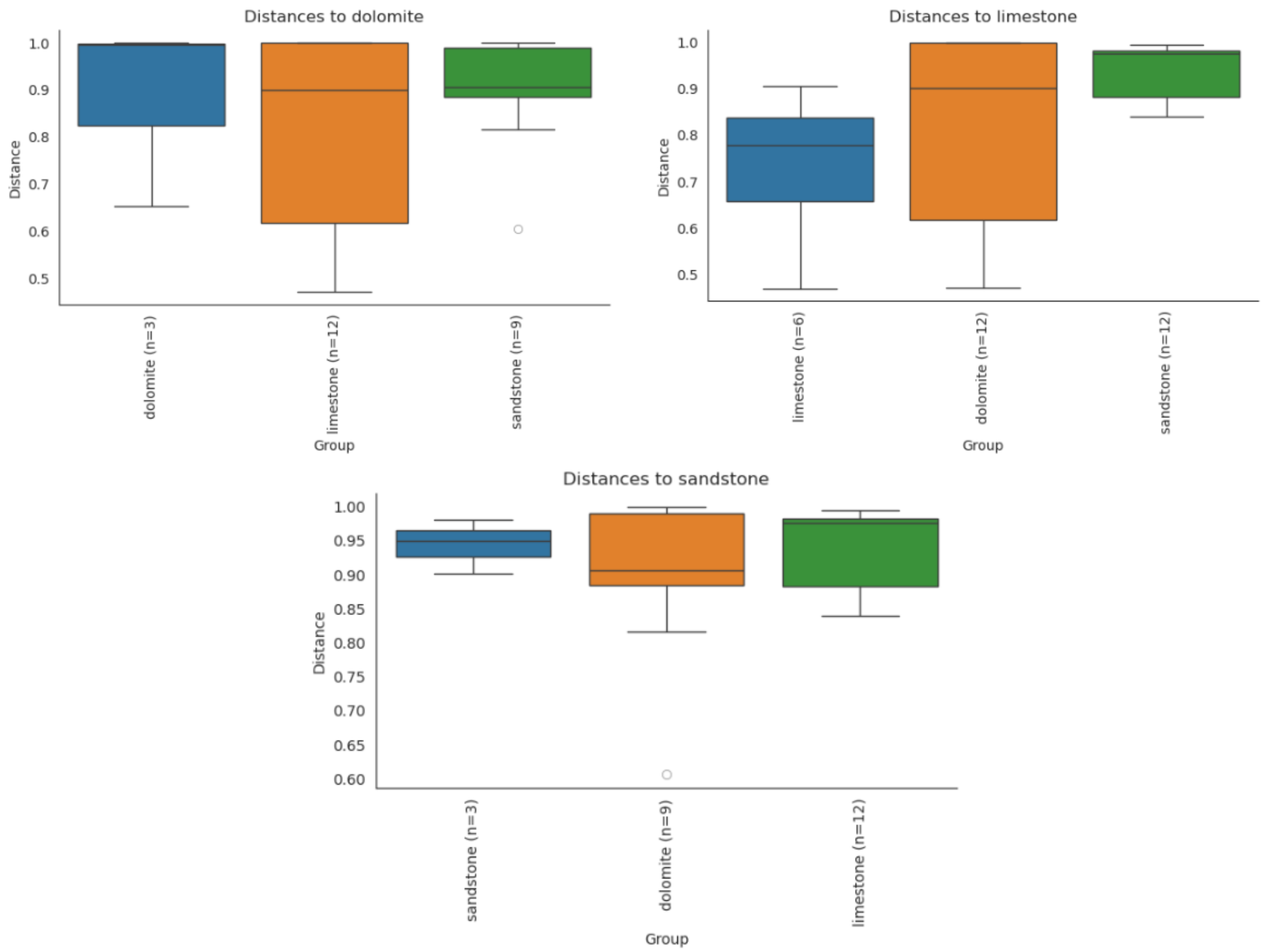


*Figure 2. Bray-Curtis dissimilarity of fungal communities across stone substrate types.*

*Tab. 4. Pairwise comparisons of fungal community composition among stone substrate types based on Bray-Curtis dissimilarity (PERMANOVA with FDR correction).*

| Group 1 | Group 2 | Sample size | Permutations | pseudo-F | p-value | q-value |
|---|---|---|---|---|---|---|
| dolomite | limestone | 7 | 999 | 1.197001 | 0.334 | 0.501 |
| | sandstone | 6 | 999 | 0.897425 | 0.503 | 0.503 |
| limestone | sandstone | 7 | 999 | 1.856716 | 0.030 | 0.090 |

*Tab. 5. Test of homogeneity of multivariate dispersion among stone substrate types based on Bray-Curtis dissimilarity (PERMDISP).*

| test statistic name | F-value |
|---|---|
| sample size | 10 |
| number of groups | 3 |
| test statistic | 0. 0.389951 |
| p-value | 0.497 |
| number of permutations | 999 |

*Tab. 6. Pairwise comparisons of multivariate dispersion among stone substrate types based on Bray-Curtis dissimilarity (PERMDISP with FDR correction).*

| Group 1 | Group 2 | Sample size | Permutations | F-value | p-value | q-value |
|---|---|---|---|---|---|---|
| dolomite | limestone | 7 | 999 | 0.162407 | 0.734 | 0.734 |
| | sandstone | 6 | 999 | 0.105868 | 0.280 | 0.420 |
| limestone | sandstone | 7 | 999 | 2.233285 | 0.143 | 0.420 |

## I.III Jaccard

*Tab. 7. Overall comparison of fungal community composition among stone substrate types based on Jaccard dissimilarity (PERMANOVA).*

| | |
|---|---|
| test statistic name | pseudo-F |
| sample size | 10 |
| number of groups | 3 |
| test statistic | 1.335105 |
| p-value | 0.109 |
| number of permutations | 999 |

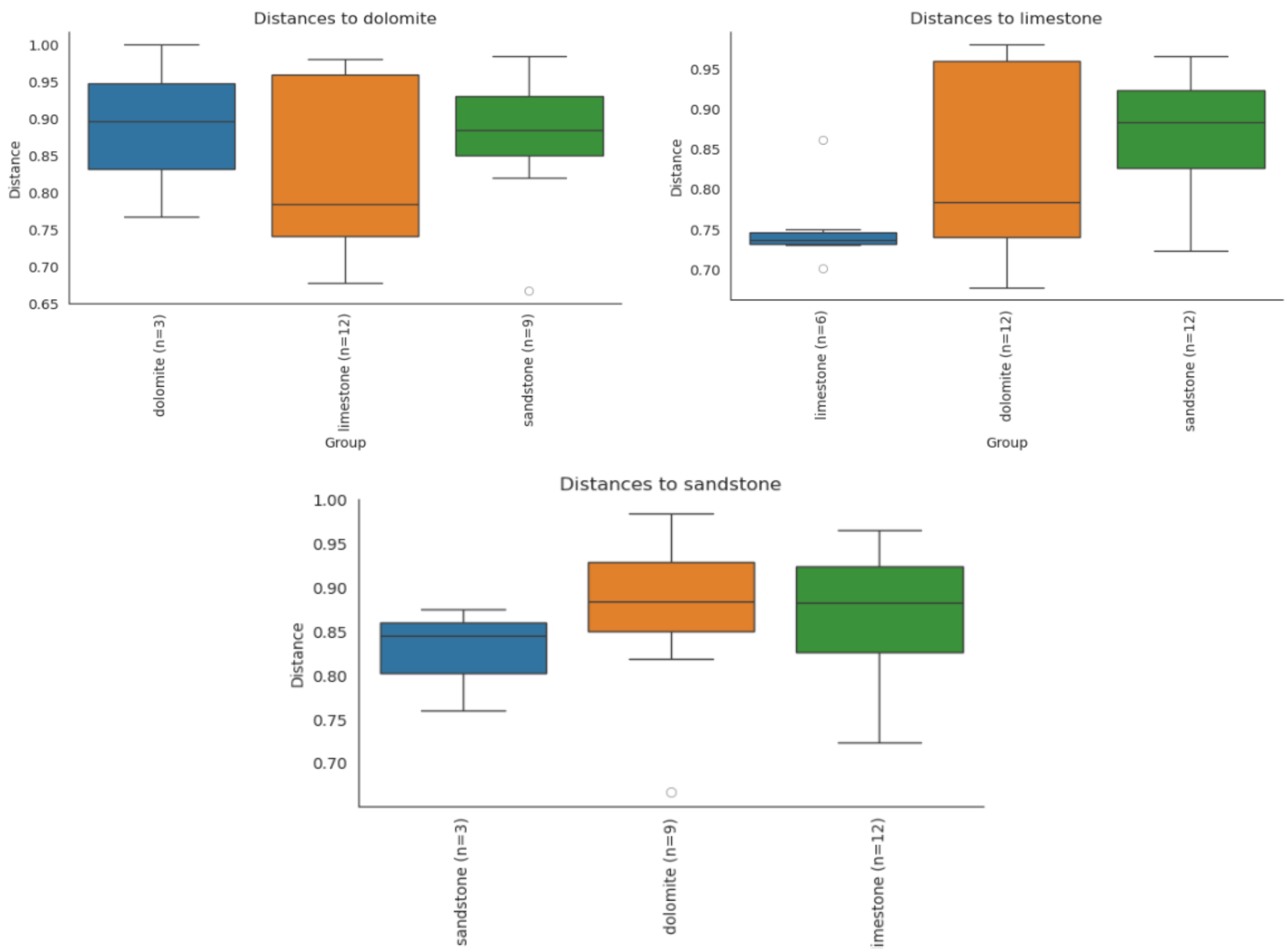


*Figure 3. Jaccard dissimilarity of fungal communities across stone substrate types.*

*Tab. 8. Pairwise comparisons of fungal community composition among stone substrate types based on Jaccard dissimilarity (PERMANOVA with FDR correction).*

| | | Sample size | Permutations | pseudo-F | p-value | q-value |
|---|---|---|---|---|---|---|
| Group 1 | Group 2 | | | | | |
| dolomite | limestone | 7 | 999 | 1.092819 | 0.298 | 0.447 |
| | sandstone | 6 | 999 | 1.141172 | 0.510 | 0.510 |
| limestone | sandstone | 7 | 999 | 1.785808 | 0.028 | 0.084 |

*Tab. 9. Test of homogeneity of multivariate dispersion among stone substrate types based on Jaccard dissimilarity (PERMDISP).*

| | |
|---|---|
| test statistic name | F-value |
| sample size | 10 |
| number of groups | 3 |
| test statistic | 0.277127 |
| p-value | 0.653 |
| number of permutations | 999 |

*Tab. 10. Pairwise comparisons of multivariate dispersion among stone substrate types based on Jaccard dissimilarity (PERMDISP with FDR correction).*

| | | Sample size | Permutations | F-value | p-value | q-value |
|---|---|---|---|---|---|---|
| Group 1 | Group 2 | | | | | |
| dolomite | limestone | 7 | 999 | 0.476118 | 0.724 | 0.736 |
| | sandstone | 6 | 999 | 0.143192 | 0.297 | 0.736 |
| limestone | sandstone | 7 | 999 | 0.134172 | 0.736 | 0.736 |

# II. Dataset: bacteria

## II.I Shannon index

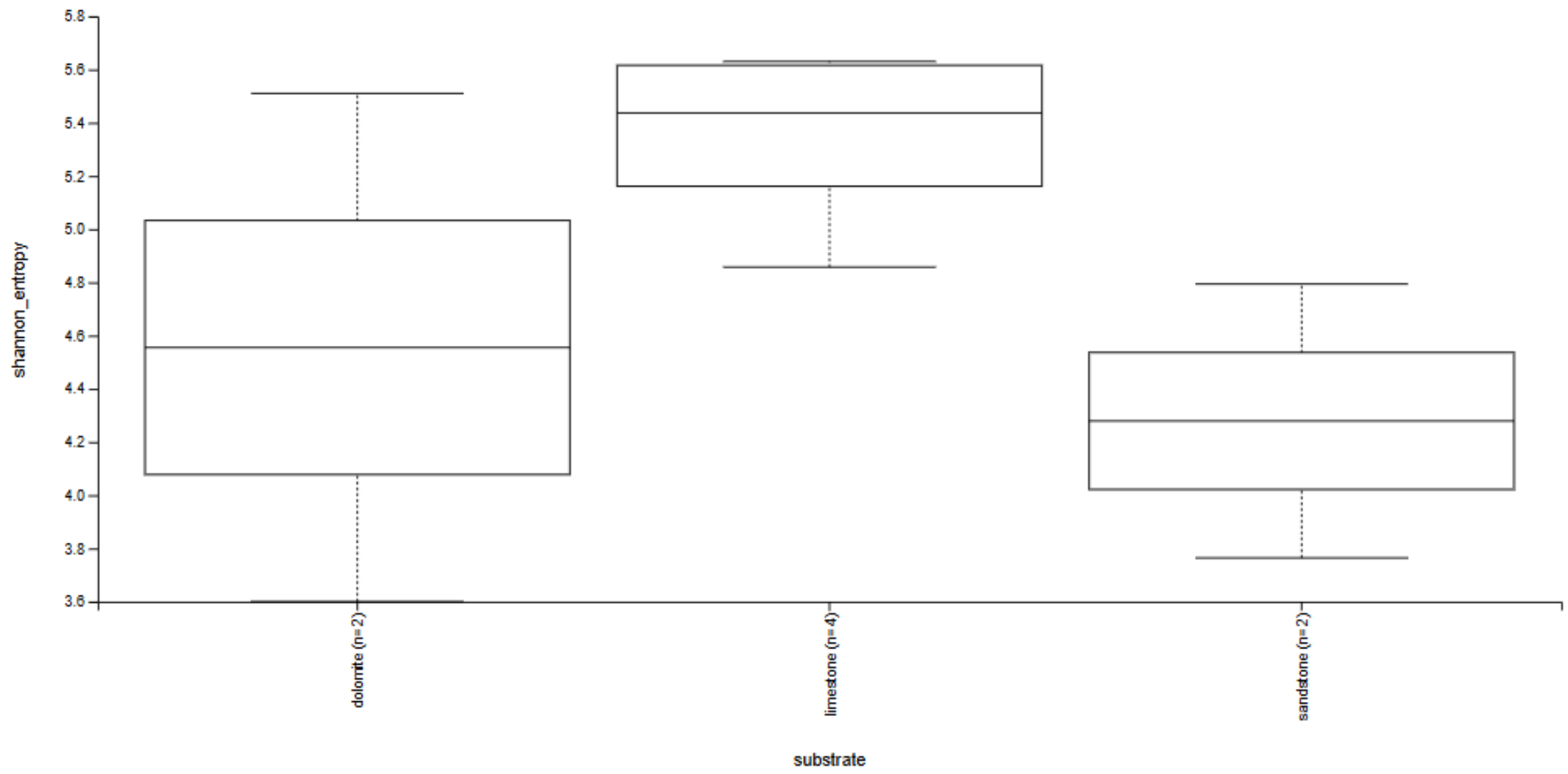


*Figure 4. Shannon diversity of bacterial communities across stone substrate types.*

*Tab. 11. Overall comparison of bacterial alpha diversity among stone substrate types based on the Shannon diversity index (Kruskal-Wallis test).*

| | Result |
|---|---|
| H | 3.1666666666666643 |
| p-value | 0.2052896575799095 |

*Tab. 12. Pairwise comparisons of bacterial alpha diversity among stone substrate types based on the Shannon diversity index (Kruskal-Wallis test with FDR correction).*

| Group 1 | Group 2 | H | p-value | q-value |
|---|---|---|---|---|
| dolomite (n=2) | limestone (n=4) | 0.857143 | 0.354539 | 0.531809 |
| | sandstone (n=2) | 0.000000 | 1.000000 | 1.000000 |
| limestone (n=4) | sandstone (n=2) | 3.428571 | 0.064078 | 0.192233 |

## II.II Bray-Curtis

*Tab. 13. Overall comparison of bacterial community composition among stone substrate types based on Bray-Curtis dissimilarity (PERMANOVA).*

| | |
|---|---|
| test statistic name | pseudo-F |
| sample size | 8 |
| number of groups | 3 |
| test statistic | 1.722997 |
| p-value | 0.001 |
| number of permutations | 999 |

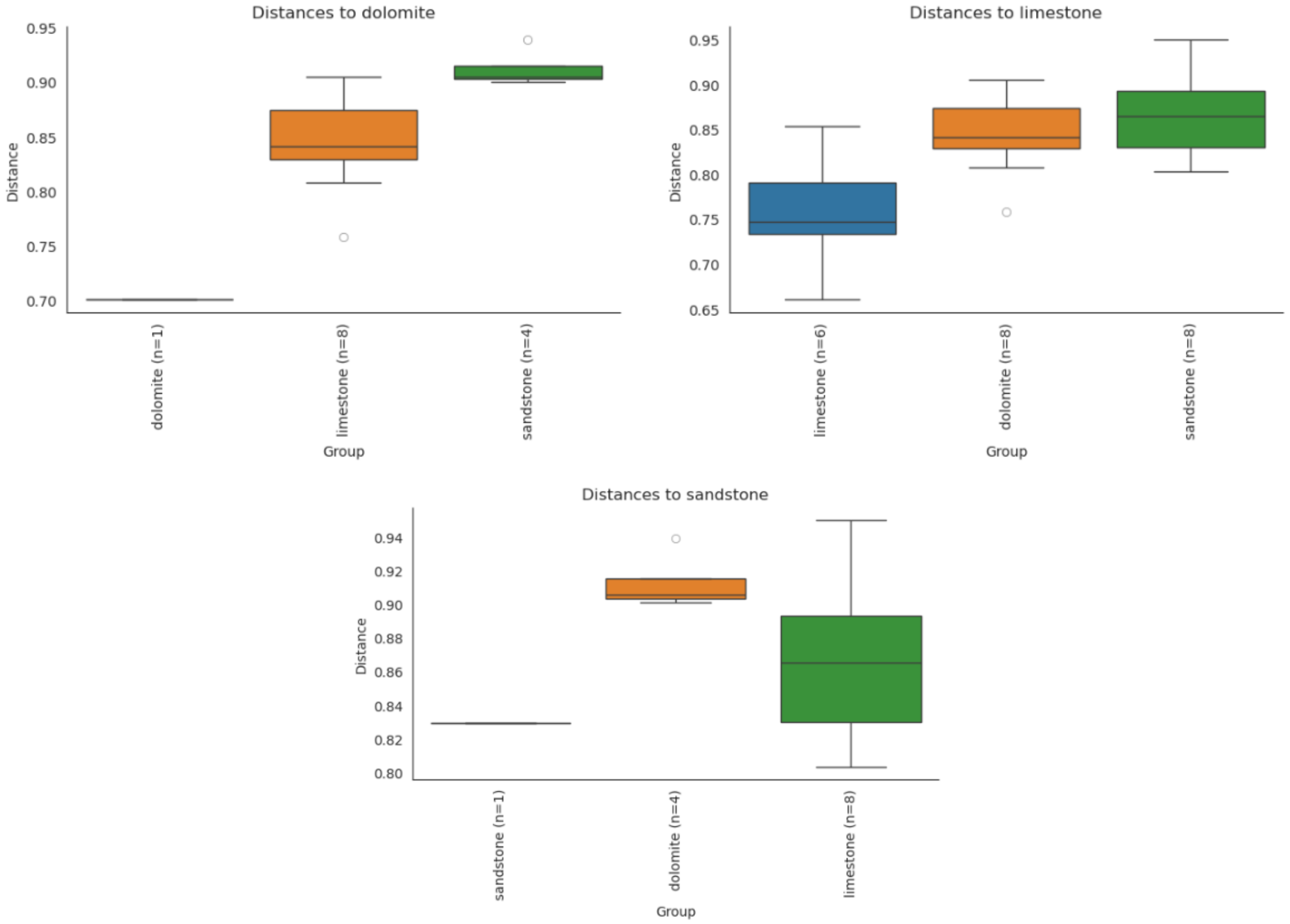


*Figure 5. Bray-Curtis dissimilarity of bacterial communities across stone substrate types.*

*Tab. 14. Pairwise comparisons of bacterial community composition among stone substrate types based on Bray-Curtis dissimilarity (PERMANOVA with FDR correction).*

| Group 1 | Group 2 | Sample size | Permutations | pseudo-F | p-value | q-value |
|---|---|---|---|---|---|---|
| dolomite | limestone | 6 | 999 | 1.788840 | 0.060 | 0.1005 |
| | sandstone | 4 | 999 | 1.830001 | 0.328 | 0.3280 |
| limestone | sandstone | 6 | 999 | 1.589774 | 0.067 | 0.1005 |

*Tab. 15. Test of homogeneity of multivariate dispersion among stone substrate types based on Bray-Curtis dissimilarity (PERMDISP).*

| test statistic name | F-value |
|---|---|
| sample size | 8 |
| number of groups | 3 |
| test statistic | 3.105267 |
| p-value | 0.641 |
| number of permutations | 999 |

*Tab. 16. Pairwise comparisons of multivariate dispersion among stone substrate types based on Bray-Curtis dissimilarity (PERMDISP with FDR correction).*

| Group 1 | Group 2 | Sample size | Permutations | F-value | p-value | q-value |
|---|---|---|---|---|---|---|
| dolomite | limestone | 6 | 999 | 4.918333 | 0.511 | 1.0 |
| | sandstone | 4 | 999 | inf | 1.000 | 1.0 |
| limestone | sandstone | 6 | 999 | 0.902997 | 0.707 | 1.0 |

## II.III Jaccard

*Tab. 17. Overall comparison of bacterial community composition among stone substrate types based on Jaccard dissimilarity (PERMANOVA).*

| | |
|---|---|
| test statistic name | pseudo-F |
| sample size | 8 |
| number of groups | 3 |
| test statistic | 1.682335 |
| p-value | 0.012 |
| number of permutations | 999 |

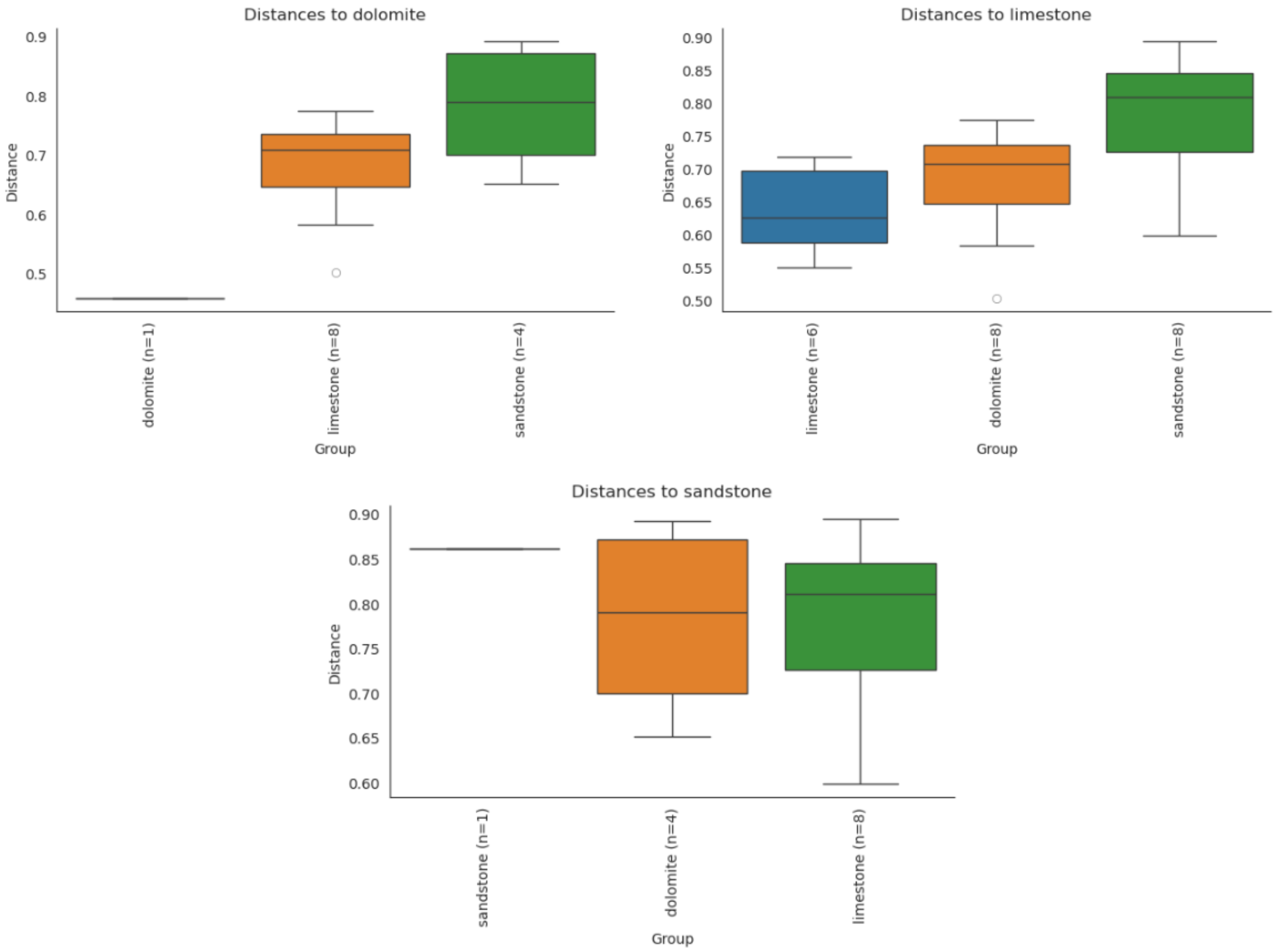


*Figure 6. Jaccard dissimilarity of bacterial communities across stone substrate types.*

*Tab. 18. Pairwise comparisons of bacterial community composition among stone substrate types based on Jaccard dissimilarity (PERMANOVA with FDR correction).*

| Group 1 | Group 2 | Sample size | Permutations | pseudo-F | p-value | q-value |
|---|---|---|---|---|---|---|
| dolomite | limestone | 6 | 999 | 1.940907 | 0.036 | 0.1080 |
| | sandstone | 4 | 999 | 1.608160 | 0.312 | 0.3120 |
| limestone | sandstone | 6 | 999 | 1.518594 | 0.143 | 0.2145 |

*Tab. 19. Test of homogeneity of multivariate dispersion among stone substrate types based on Jaccard dissimilarity (PERMDISP).*

| test statistic name | F-value |
|---|---|
| sample size | 8 |
| number of groups | 3 |
| test statistic | 6.729266 |
| p-value | 0.128 |
| number of permutations | 999 |

*Tab. 20. Pairwise comparisons of multivariate dispersion among stone substrate types based on Jaccard dissimilarity (PERMDISP with FDR correction).*

| Group 1 | Group 2 | Sample size | Permutations | F-value | p-value | q-value |
|---|---|---|---|---|---|---|
| dolomite | limestone | 6 | 999 | 7.648460 | 0.061 | 0.183 |
| | sandstone | 4 | 999 | inf | 0.673 | 0.734 |
| limestone | sandstone | 6 | 999 | 0.552101 | 0.734 | 0.734 |

# III. Dataset: phototrophs

## III.I Shannon index

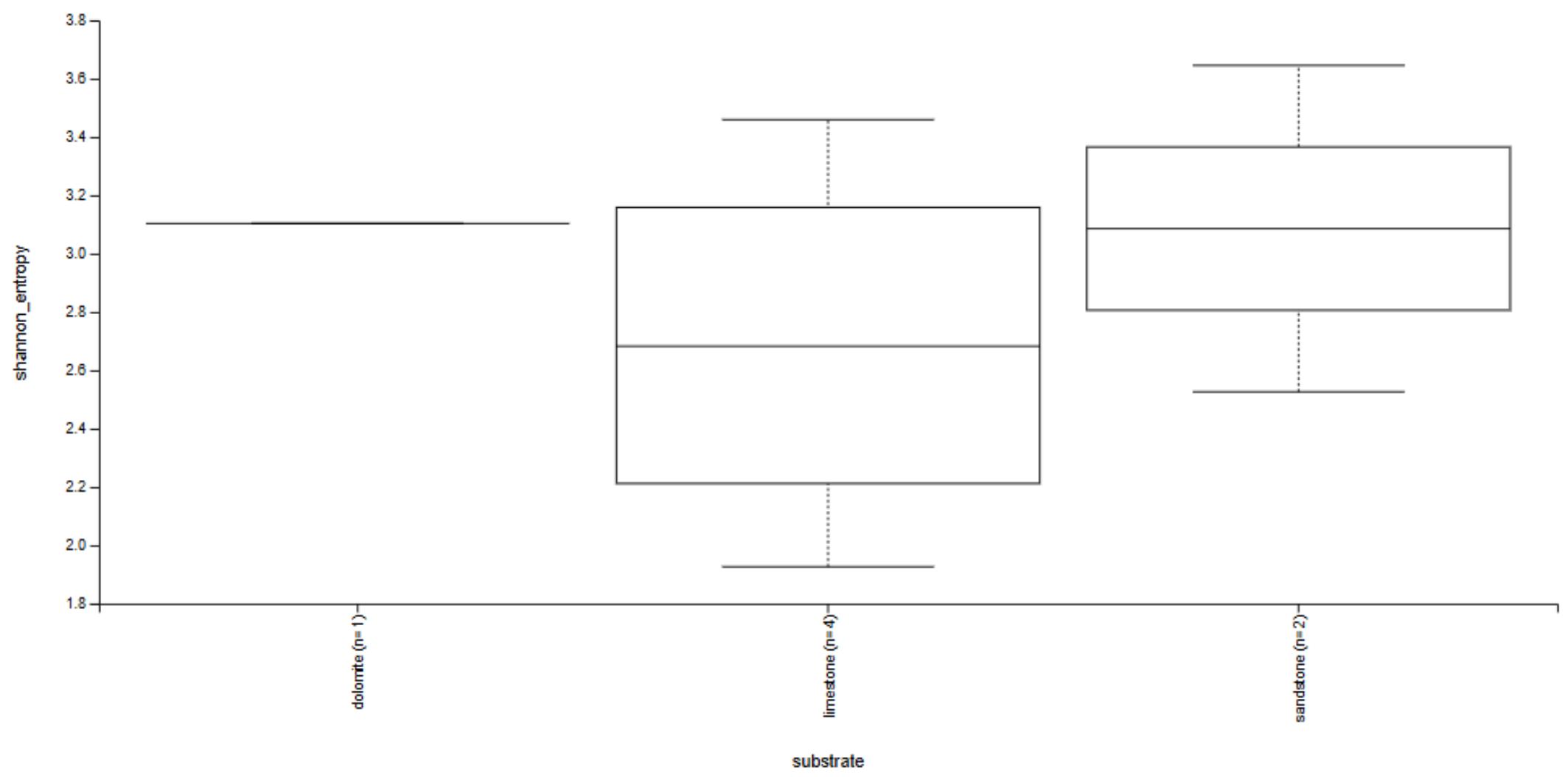


*Figure 7. Shannon diversity of phototrophic communities across stone substrate types.*

*Tab. 21. Overall comparison of phototrophic alpha diversity among stone substrate types based on the Shannon diversity index (Kruskal-Wallis test).*

| | Result |
|---|---|
| H | 1.125 |
| p-value | 0.569782824730923 |

*Tab. 22. Pairwise comparisons of phototrophic alpha diversity among stone substrate types based on the Shannon diversity index (Kruskal-Wallis test with FDR correction).*

| Group 1 | Group 2 | H | p-value | q-value |
|---|---|---|---|---|
| dolomite (n=1) | limestone (n=4) | 0.500000 | 0.479500 | 0.71925 |
| | sandstone (n=2) | 0.000000 | 1.000000 | 1.00000 |
| limestone (n=4) | sandstone (n=2) | 0.857143 | 0.354539 | 0.71925 |

## III.II Bray-Curtis

*Tab. 23. Overall comparison of phototrophic community composition among stone substrate types based on Bray-Curtis dissimilarity (PERMANOVA).*

| | |
|---|---|
| test statistic name | pseudo-F |
| sample size | 7 |
| number of groups | 3 |
| test statistic | 1.306177 |
| p-value | 0.105 |
| number of permutations | 999 |

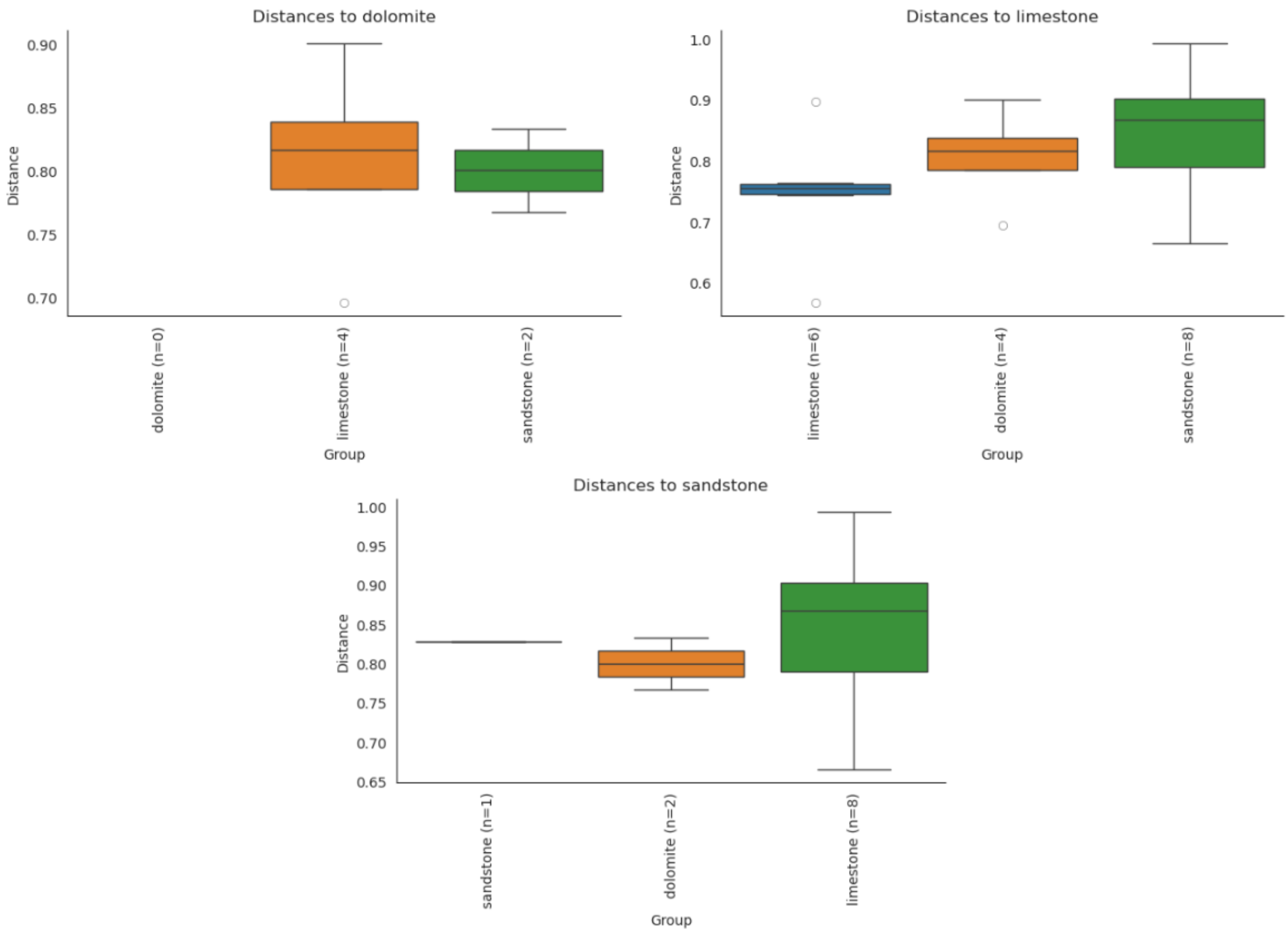


*Figure 8. Bray-Curtis dissimilarity of phototrophic communities across stone substrate types.*

*Tab. 24. Pairwise comparisons of phototrophic community composition among stone substrate types based on Bray-Curtis dissimilarity (PERMANOVA with FDR correction).*

| Group 1 | Group 2 | Sample size | Permutations | pseudo-F | p-value | q-value |
|---|---|---|---|---|---|---|
| dolomite | limestone | 5 | 999 | 1.250656 | 0.196 | 0.294 |
| | sandstone | 3 | 999 | 0.915217 | 0.670 | 0.670 |
| limestone | sandstone | 6 | 999 | 1.531751 | 0.193 | 0.294 |

*Tab. 25. Test of homogeneity of multivariate dispersion among stone substrate types based on Bray-Curtis dissimilarity (PERMDISP).*

| | |
|---|---|
| test statistic name | F-value |
| sample size | 7 |
| number of groups | 3 |
| test statistic | 8.00341 |
| p-value | 0.892 |
| number of permutations | 999 |

*Tab. 26. Pairwise comparisons of multivariate dispersion among stone substrate types based on Bray-Curtis dissimilarity (PERMDISP with FDR correction).*

| Group 1 | Group 2 | Sample size | Permutations | F-value | p-value | q-value |
|---|---|---|---|---|---|---|
| dolomite | limestone | 5 | 999 | 11.745111 | 1.000 | 1.0 |
| | sandstone | 3 | 999 | inf | 1.000 | 1.0 |
| limestone | sandstone | 6 | 999 | 0.205159 | 0.707 | 1.0 |

## III.III Jaccard

*Tab. 27. Overall comparison of phototrophic community composition among stone substrate types based on Jaccard dissimilarity (PERMANOVA).*

| | |
|---|---|
| test statistic name | pseudo-F |
| sample size | 7 |
| number of groups | 3 |
| test statistic | 0.855796 |
| p-value | 0.749 |
| number of permutations | 999 |

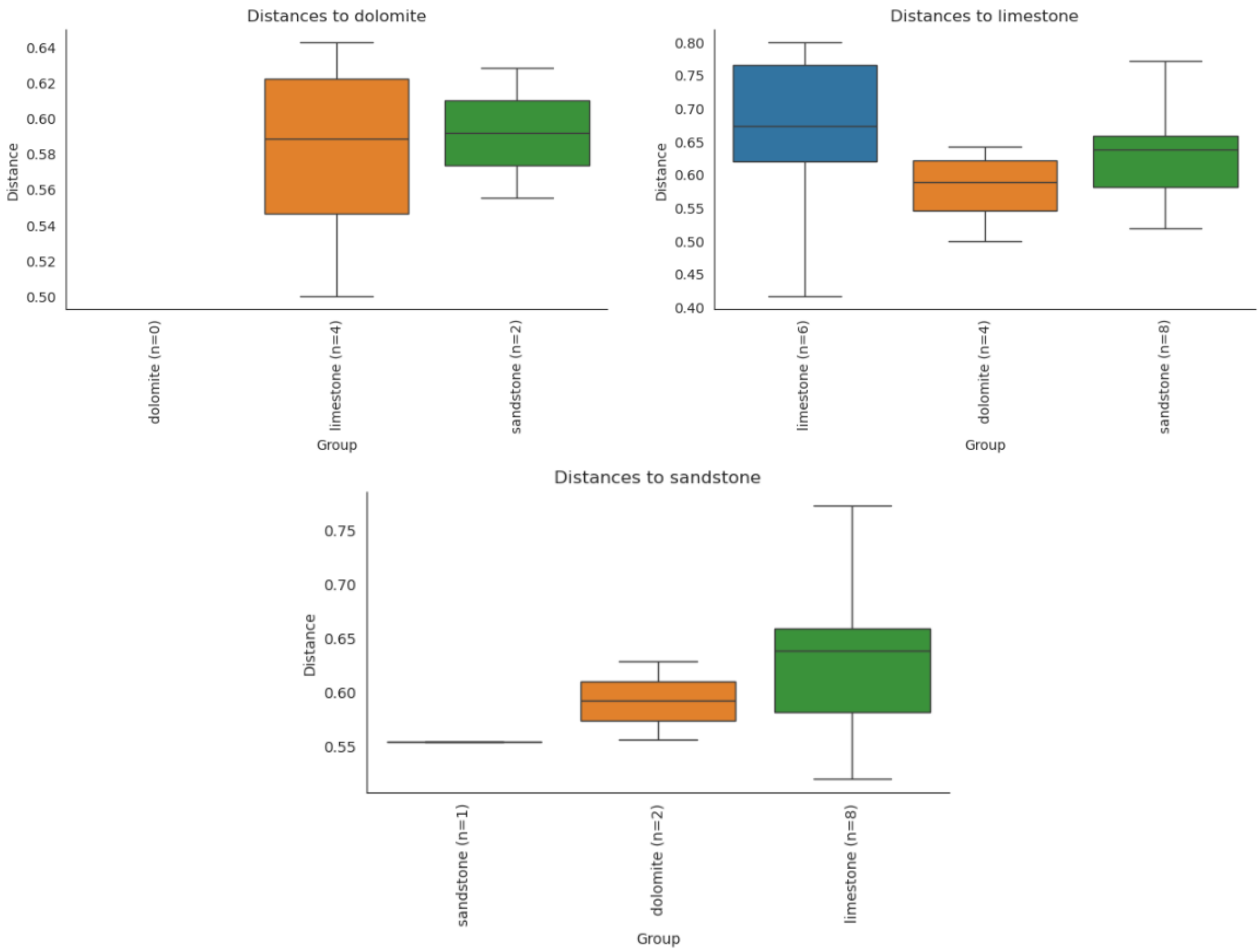


*Figure 9. Jaccard dissimilarity of phototrophic communities across stone substrate types.*

*Tab. 28. Pairwise comparisons of phototrophic community composition among stone substrate types based on Jaccard dissimilarity (PERMANOVA with FDR correction).*

| Group 1 | Group 2 | Sample size | Permutations | pseudo-F | p-value | q-value |
|---|---|---|---|---|---|---|
| dolomite | limestone | 5 | 999 | 0.599514 | 1.000 | 1.000 |
| | sandstone | 3 | 999 | 1.197670 | 0.357 | 0.783 |
| limestone | sandstone | 6 | 999 | 1.012541 | 0.522 | 0.783 |

*Tab. 29. Test of homogeneity of multivariate dispersion among stone substrate types based on Jaccard dissimilarity (PERMDISP).*

| | |
|---|---|
| test statistic name | F-value |
| sample size | 7 |
| number of groups | 3 |
| test statistic | 3.193127 |
| p-value | 0.975 |
| number of permutations | 999 |

*Tab. 30. Pairwise comparisons of multivariate dispersion among stone substrate types based on Jaccard dissimilarity (PERMDISP with FDR correction).*

| Group 1 | Group 2 | Sample size | Permutations | F-value | p-value | q-value |
|---|---|---|---|---|---|---|
| dolomite | limestone | 5 | 999 | 4.700585 | 1.000 | 1.0 |
| | sandstone | 3 | 999 | inf | 1.000 | 1.0 |
| limestone | sandstone | 6 | 999 | 0.959213 | 0.599 | 1.0 |
